# Decomposing the neurocomputational mechanisms of deontological moral preferences

**DOI:** 10.1093/pnasnexus/pgag074

**Published:** 2026-04-07

**Authors:** Yoonseo Zoh, Soyeon Kim, Hackjin Kim, M J Crockett, Woo-Young Ahn

**Affiliations:** Department of Psychology, Princeton University, Princeton, NJ, USA; Department of Psychology, Seoul National University, Seoul 08826, Korea; School of Psychology, Korea University, Seoul, Korea; Department of Psychology, Princeton University, Princeton, NJ, USA; University Center for Human Values, Princeton University, Princeton, NJ, USA; Department of Psychology, Seoul National University, Seoul 08826, Korea; Department of Brain and Cognitive Sciences, Seoul National University, Seoul, Korea; AI Institute, Seoul National University, Seoul, Korea

## Abstract

Research on the neurocomputational mechanisms of moral judgment has typically focused on contrasting “utilitarian” preferences to impartially maximize aggregate welfare and “deontological” preferences that judge the morality of actions based on rules. However, there has been little work to decompose the cognitive subcomponents of deontological preferences. Here, we investigated the neurocomputational mechanisms underlying two types of deontological preferences (Rawlsian and Kantian) and their contrast with utilitarian preferences in an incentivized moral dilemma task. Participants repeatedly decided how to allocate harm between a single individual (“the one”) and a group of three to four individuals (“the group”). The task distinguished preferences for Rawlsian, Kantian, and utilitarian strategies by quantifying trade-offs among active harm, concern for the worst-off individual, and overall utility. Behaviorally, participants favored the Rawlsian strategy, preferring to impose more harm overall rather than disproportionately harm the one individual. Computational modeling revealed two dissociable dimensions of individual variability in Rawlsian preferences: (i) minimizing the maximum amount of harm delivered to a single person and (ii) subjective threshold of acceptable amount of harm imposed on one person. The combination of univariate and multivariate functional MRI analyses revealed the engagement of distinct brain regions in these two dimensions of Rawlsian preferences, which respectively mapped onto activity in mentalizing and valuation networks. Our results reveal the neurocomputational mechanisms guiding trade-offs between the welfare of one versus a larger group and highlight distinct roles for the mentalizing and valuation networks in shaping Rawlsian moral preferences.

Significance Statement Moral dilemmas often pit harm to one person against the welfare of many. Here, we examined whether choosing to protect an individual in such a scenario reflects a single process or arises from multiple mechanisms. Combining computational modeling with neuroimaging, we show that concern for harming an individual separates into two distinct dimensions: minimizing maximum harm to an individual and setting a threshold for acceptable harm. These dimensions drove distinct patterns of moral choice and engaged separable neural regions. Our findings demonstrate that the moral concern about harming an individual is multidimensional, uncovering the neurocomputational mechanisms by which people weigh consideration for individuals against the welfare of the group.

## Introduction

Many common moral dilemmas raise the question of whether it is justifiable to impose sacrifices on some people to increase overall welfare (1). Moral judgments on this issue mark critical distinctions between different moral principles and have occupied a central place in the moral psychology literature (2–5). For example, the classic “trolley dilemma” of whether to sacrifice one person to save five put deontological and utilitarian principles in dispute by presenting the option of taking a harmful action, which is categorically prohibited, as the only means to achieve the better outcome (6, 7). Observations of people's responses to such sacrificial dilemmas led to the dual process model, which mapped the tension between deontology and utilitarianism onto intuitive and deliberative processes (2, 8–11).

The dominant responses against harming one person in a sacrificial dilemma reflect a categorical (deontic) prescription in Kantian ethics, which, however, only accounts for a fraction of deontological theories. Actual moral decisions made by lay people are unlikely to be fully captured by the unconditional prohibition imposed by rule-based Kantian ethics. There is more to the deontological principle concerning justice and fairness and its tension with consequentialism. It is possible that fairness-based deontological ethics focused on mutual understanding and commitment to cooperation may better explain people's moral decisions and their psychological underpinnings from a normative standpoint (5, 12–14).

The Rawlsian approach is another deontological principle that reflects concerns for justice by promoting inviolable individual rights and fair divisions of benefits and burdens. John Rawls, in opposition to utilitarianism, argued “justice denies that the loss of freedom for some is made right by a greater good shared by others” (15). The Rawlsian principle requires that the prospects of the worst-off be maximized, ensuring agreement among all parties from an impartial standpoint and that this evaluation take precedence over considerations of efficiency. While a Rawlsian approach to deontological ethics has received comparatively less attention in understanding our moral decisions, scenarios that pit utilitarian against the Rawlsian principles have shown an overall preference for Rawlsian approach, suggesting its accordance with commonly held moral views of lay people (5, 12, 16–19). For example, when a suggested policy incurs costs on some individuals, people object to implementing the policy even though doing so will lead to an overall increase in welfare (16).

Despite the common observation of preference for the Rawlsian approach, however, we know little about the motivations or computations driving decisions that conflict with moral principles outside of strict deontic rules or welfare maximization. Also, what underlies individual differences in preference of a decision that is aligned with Rawlsian principle over other normative moral principles is poorly understood. Consequently, mapping between moral decisions predicted by normative ethical principles and their underlying psychological dimensions has likely been incomplete.

One clue in understanding people's preferences for the Rawlsian approach comes from research on the singularity effect. Previous work on empathy showed people are strongly motivated to focus on protecting the mishap of a single individual, but such motivation fails to extend to a large number of people in need (20–24). For example, an iconic photograph of a single child suffering from the refugee crisis evoked great attention and donations of money, which, in contrast, failed to be elicited by statistics of hundreds of thousands death toll (25). Even in the scenarios where the victim is not identifiable, people cared about saving the individual victim much more than saving a group of victims, suggesting people's insensitivity to the absolute number of lives at risk (20, 26–28).

A neuroimaging study has suggested such preferences toward protecting a single, individual victim are imposed by the unique characteristic of our mentalizing ability, being able to represent the mind of other individuals only one at a time (29). It is possible that Rawlsian moral preference (which emphasizes improving the situation of the worst-off person) may share a common psychological underpinning with the singularity effect, as people weight the worst-off individuals’ suffering more than that of others. Mentalizing biased toward the worst-off individual, but not the group as a whole, may guide a strong motivation to contribute to helping one person, even at the expense of aggregate utility.

Previous work also demonstrated the role of mentalizing in Rawlsian preference. The Rawlsian strategy of maximizing the “worst-off” position as reflected in its “maximin” principle in distributive decisions (maximize the prospect with respect to the least advantaged position) was shown to correlate with the activity in mentalizing regions including the right temporoparietal junction (TPJ) (30). Notably, this Rawlsian preference in distributive decisions for others was concordant with avoiding the worst possible outcome in gambling decisions for self at the behavioral and neural levels, suggesting a domain-general avoidance of the worst-off outcome driven by its magnified representation in mentalizing networks (30). However, the role of mentalizing networks in decisions to protect the worst-off individual from harm has not been tested, and it remains unknown whether mentalizing networks are also implicated in computations in pitting Rawlsian principle (maximizing the minimum welfare; maximin) against utilitarian principle (minimizing the aggregated harm) in allocation of harm.

Another psychological dimension that might be critical in explaining the psychological basis of Rawlsian preference is the representation of agreeability. The Rawlsian principle is grounded on the contractualist idea that the morality of an action is founded upon the mutual agreement of all involved parties (15, 31–33). Rawls, by introducing the idea of the original position where no one knows in advance where they will end up being situated in the society, aimed to establish a theory of justice that is beneficial and fair to all—and therefore everyone would reasonably agree to choose (15). While it is unlikely that people apply an impartial reasoning in a strict sense as Rawls conceived, people may spontaneously adopt the idea of agreeability and determine whether the option is acceptable to all in guiding their moral decisions (34). Therefore, we hypothesized that the representation of agreeability might be another important dimension of Rawlsian moral preference that is separable from the biased mentalizing toward the worst-off individual.

Consistent with this hypothesis, prior studies have found evidence of contractualist moral intuitions in lay people's moral reasoning (14, 34, 35). We examined whether such contractualist moral intuition composes a meaningful psychological dimension of Rawlsian moral preference. In psychological space, the idea of agreeability may be translated into a signature of differing interpretations across individuals on what is a fair outcome to all. While this may not be easy to capture with behavioral data alone, at a computational level, we expected it to be quantifiable as subjective thresholds of what a decision-maker considers a reasonable distribution of the benefits and burdens from an impartial standpoint. Therefore, we sought to identify a computational signature of contractualist intuition in Rawlsian moral preference as well as its neural instantiation, which we posited to be psychologically and neurally distinct from the heightened concern for an individual in the worst-off position.

**Figure 1:**
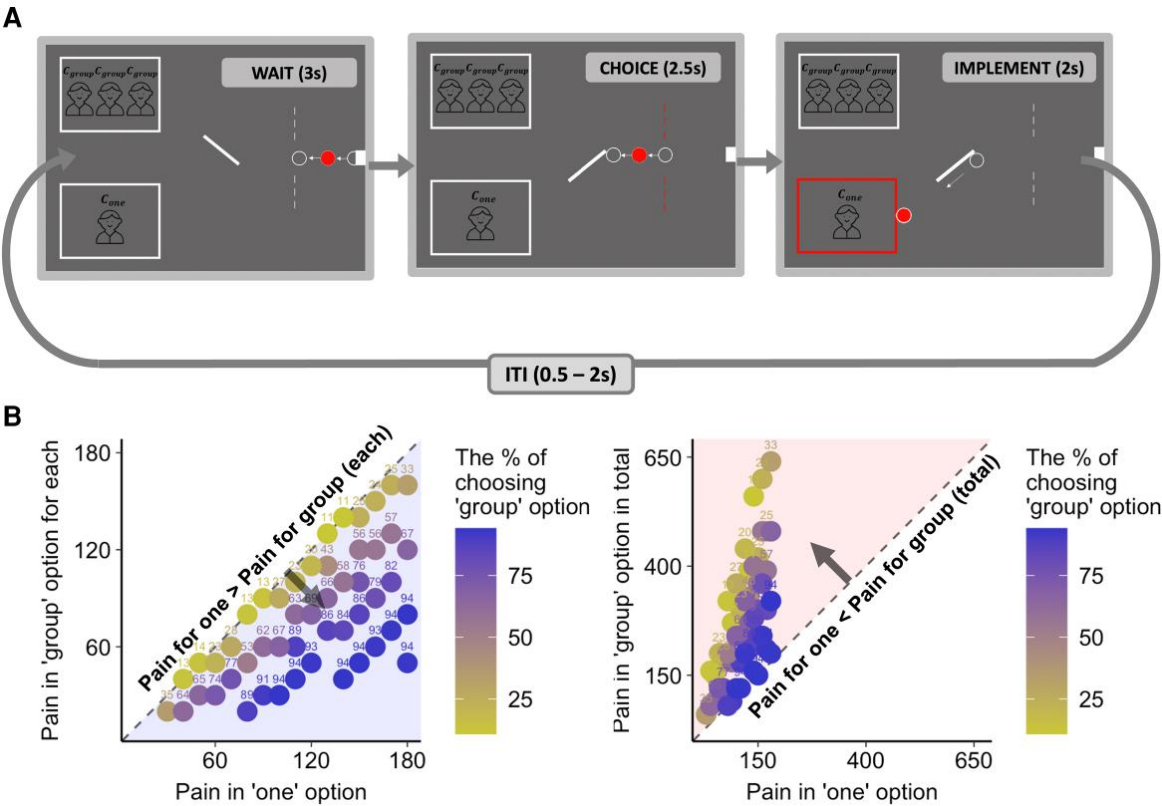
Experimental design and choice behavior. A) Schematic representation of the harm allocation task. Participants repeatedly chose how to allocate harm between a single individual and a group of three to four individuals. Here, the amount of harm was specified as the length of time getting a cold-pressor test and shown on the top of each person icon representing individuals. A trial starts with a red ball proceeding across the screen toward two different options. The direction of the lever in the middle indicates the default option that will be implemented if the participant took no action (wait stage). After the ball crosses the dotted line, the participant may press the button to switch the lever and direct the ball toward the other option (choice stage). Once the ball hits the lever, the participant can no longer switch it. And the ball continues to move toward the option the participant has chosen to assign the harm and then the box surrounding the chosen option changes color once the ball hits it (implement stage). B) Preference for the Rawlsian option as a function of harm for one versus group. Across trials, the amount of harm delivered to the one person and the group of people varied. Each dot represents a unique combination of trial parameters. The left plot compares pain delivered to the one option versus each individual in the group option, while the right plot compares pain delivered to the one option versus the total pain across all individuals in the group option. By design, minimizing the total amount of harm always required delivering at least an equal or greater amount of harm to the one than to each member of the group. Participants therefore had to choose between selecting a utilitarian option (minimize the total amount of harm delivered) or a Rawlsian option (minimizing the maximum amount of harm delivered to a single individual). Preference for the Rawlsian option increased with increasing harm delivered to the one individual. The gray dotted line indicates equivalent amounts of harm for the one and the group.

To this end, we deployed a novel functional MRI (fMRI) paradigm where participants had to repeatedly decide how to allocate harm between a single individual (“the one”) and a group of three to four individuals (“the group”; Fig. 1A). We operationalized harm as an amount of time spent completing a cold-pressor test (Bayer et al., 2005). We manipulated the amount of harm in the one option to be always the same or bigger than the amount of harm allocated to each individual in the group option $\left(C_{\mathrm{one}}\right)$ but was always the same or smaller than the total amount of harm involved in “the group” option ($C_{\mathrm{group}}\times N_{\mathrm{group}}$; Fig. 1B). There were 50 unique combinations of trials, each repeated three times under different default conditions (no default, default allocation to the one, and default allocation to the group), making 150 trials in total.

With this task, we were able to capture the degree to which individuals take into account different normative considerations in harm allocation decisions. In particular, the task enabled us to characterize how much one upholds a Rawlsian strategy over a utilitarian strategy when they are pitted against each other. Furthermore, we were able to distinguish Rawlsian from Kantian preferences by enabling participants on a subset of trials to either make an active choice or allow a default outcome to occur, revealing the degree to which participants preferred to avoid performing harmful actions irrespective of outcomes. By applying computational models to choice behavior, we examined computations involved in this trade-off as well as whether they can be ascribed to distinct psychological dimensions of Rawlsian preferences. We hypothesized (i) preferences for the maximin strategy and (ii) representation of agreeability.

We characterized the neural basis of these two dimensions using univariate and multivariate methods. Importantly, the motivation to protect a worst-off individual manifested as maximin strategy is directional in a sense that employing more of the strategy means the individual prefers the Rawlsian principle over the utilitarian principle. However, the dimension of agreeability has no such directional orientation, because giving more or less to a single individual does not necessarily mean being more committed to Rawlsian principle but represents a different idea of agreeability one holds. Therefore, for the maximin dimension, we applied a univariate model-based approach for identifying brain regions that scale with the recruitment of the maximin strategy. On the other hand, for probing the neural instantiation of the agreeability dimension, the multivariate approach of intersubject representational similarity analysis (IS-RSA) was applied to examine which brain region shows shared responses among participants who have a similar idea of agreeability. We specifically examined whether participants with similar profiles of agreeability threshold also exhibit similar neural representations of harm concerning an individual recipient during moral decision-making. Our analysis revealed the neural encoding patterns of harm that are similar among participants who shared a similar level of agreeability, independent of the actual decisions made.

The utilization of both univariate and multivariate neuroimaging analysis techniques enabled us to investigate the neural mappings associated with two distinct dimensions of Rawlsian moral principles. Our research findings shed light on the psychological dimensions that underlie Rawlsian moral preferences, offering new perspectives into the moral intuitions that shape individuals’ judgments when weighing different moral principles.

## Results

### The Rawlsian strategy was favored in harm allocation decisions

Our task design was able to distinguish three different normative moral preferences: Rawlsian, Kantian, and utilitarian. Rawlsian and utilitarian preferences were distinguished by choices between the one and the group options. The one option entailed less harm overall than the group option. Therefore, the choice between two options pits the maximin (maximizing the minimum welfare—in the context of our task, this means minimizing the maximum amount of harm) Rawlsian strategy against the utilitarian strategy of maximizing the overall welfare, which means minimizing the overall amount of harm in our task, with the choice of the group indicating one favored the Rawlsian strategy over the utilitarian strategy.

We first examined the proportion of trials on which participants chose the Rawlsian strategy over the utilitarian strategy. Most participants favored the Rawlsian option (the group), with the proportion of Rawlsian choice significantly different from chance (*M* = 0.59, SD = 0.20, *t*(67) = 3.68, *P* < 0.001). Moreover, when comparing the total amount of harm participants chose to assign to the one versus the group, participants chose to give 68.36 s (SD = 37.19) of more harm overall to the group on average in order to save an individual from being disproportionately targeted.

Next, we examined the prevalence of Kantian preferences. Kantian ethics posits that certain actions should be absolutely prohibited. In the context of our task, making an active choice of who gets harmed is wrong, because harming is morally not permissible at any cost. Therefore, Kantian preferences should bias participants toward “inaction” or allowing the default option to be implemented without any active choice. In our task design, we repeated the same choice set across three conditions that differed in terms of default option (“one default,” “group default,” and “no default”), and thus, we were able to compare participants’ choices across conditions and test whether participants indeed displayed Kantian preference toward allowing a default option.

**Figure 2:**
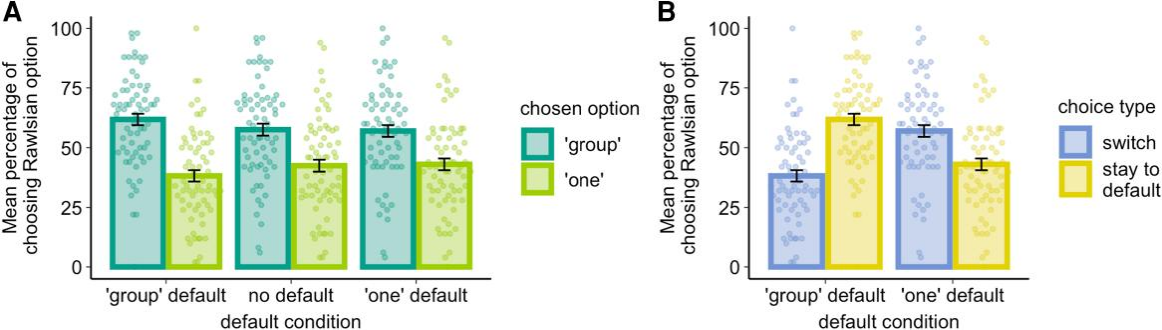
Rawlsian preferences dominate utilitarian and Kantian preferences. A) In the no-default condition, most participants preferred the Rawlsian option over the utilitarian one. We observed a significant increase in preference for the default option when the Rawlsian option was presented as the default choice. That is, the Rawlsian option was more likely to be chosen when it was presented as default, compared with when there was no-default option or when the Rawlsian option was presented as the alternative option, which provides partial support for the moral preference predicted by the Kantian ethics. B) The impact of default condition, however, was qualified by participants’ choice preference. There was a strong preference for the Rawlsian option regardless of whether it was presented as a default or the alternative option.

We do not find evidence for an overall Kantian preference, as participants were not more likely to choose the default option over the active choice across conditions (Fig. 2: the difference in probability of switching to the alternative option between one-default and group-default conditions: *t*(67) = 3.99; *P* < 0.001). Instead, we observed that the preference for the default option was evident only when the default option aligned with the Rawlsian preferences, as participants were more likely to choose the Rawlsian option when it was presented as the default (Fig. 2, difference in choice rate for the Rawlsian option across three conditions: *F*(1.45, 97.05) = 14.56; *P* < 0.001: difference in choice rate for Rawlsian option between group-default and no-default conditions: *t*(67) = 4.03, *P* < 0.001; difference in choice rate for the Rawlsian option between group-default and one-default conditions: *t*(67) = 4.13, *P* < 0.001). This suggests that Rawlsian option was strongly favored over the utilitarian option irrespective of whether it was presented as a default or alternative. In other words, Rawlsian preference to avoid disproportionately harming the worst-off individual overruled consideration of inaction of active harm as prescribed by Kantian ethics.

### Computational model of harm allocation decisions

We next built a set of computational models to examine what computations go into harm allocation decisions and the sources of individual variability in Rawlsian moral preferences. Our models were theoretically motivated with respect to two dimensions of Rawlsian moral preference we hypothesized. Firstly, we modeled the motivation to protect a worst-off individual or the maximin strategy, where the utility comparison is made for the worst-off outcome for an individual in two options. This Rawlsian strategy was directly pitted against the utilitarian strategy of “minimizing aggregate harm” in our task, where the utility comparison is made between the total amounts of harm between the two options. Therefore, we considered a weighting parameter, noted as *α* (alpha), indicating how much participants gave weight to the welfare of the one versus the group.

Next, we considered how people might take into account agreeability in harm allocation decisions, which we hypothesized to be a distinct dimension of Rawlsian moral preference from the maximin. Therefore, we considered a parameter that reflects the representation of agreeability and is subject to the employment of the maximin strategy. This parameter, noted as *φ* (phi), expressed a participant's subjective threshold of (reasonably) acceptable amount of harm to impose on one person. We conducted parameter estimation and model comparison using a hierarchical Bayesian approach (36–38). The model that best explained our choice data, as indicated by the smallest leave-one-out information criterion (LOOIC) value (39), included three free parameters: (i) a maximin parameter bounded between 0 and 1, (ii) an agreeability intercept (constant term), and (iii) an inverse temperature parameter. This model accurately predicted choice behavior in our task, outperforming a range of alternative models (see Table S1 for alternative models we compared; Fig. S1, correlation between model prediction and observed choice proportion of Rawlsian decision: *r* = 0.99, *P* < 0.001).

Our best model posits the difference in subjective utility between the Rawlsian option and the utilitarian option is computed as weighted differences between the two strategies. The outperformance of this model over the ones that constrained the weight to either 0 or 1 (*α* constrained to 1; difference in expected predictive accuracy: −2,144.2, SE = 195.3; *α* constrained to 0; difference in expected predictive accuracy: −3,438.5, SE = 191.4) indicates that people balanced the utilitarian and Rawlsian moral considerations in harm allocation decisions, rather than only relying on one strategy. Additionally, inclusion of an agreeability parameter markedly increased the model fit to data (difference in expected predictive accuracy: −270.8, SE = 43.8), suggesting the representation of agreeability indeed comprises a psychologically distinct dimension of Rawlsian moral preference. The agreeability parameter, by being adjoined to maximin computation, was able to capture what participants considered a reasonably acceptable amount of harm to allocate more or less to an individual.

Next, we looked into the estimated values of *α* and *φ*, two free parameters capturing individual variability in Rawlsian moral preference. The estimated value of parameter *α* at the group level was 0.95 (SD = 0.01). This confirms that participants heavily relied on the Rawlsian maximin strategy more than the utilitarian strategy in their choice, placing a higher priority on saving an individual from the worst-off position than on maximizing the total utility. On the other hand, unlike the *α* parameter which had lower variance with a strong preference toward the Rawlsian strategy, individual estimates of the *φ* parameter showed a wide range of values (the estimated value at the group level: *M* = −10.03, SD = 2.85), from negative to positive, suggesting substantial individual variability in the subjective threshold of an acceptable amount of more or less burden for an individual. Importantly, there was a weak, nonsignificant correlation between the estimated parameter value of *α* and φ (Fig. S3, *r*(66) = 0.13, *P* = 0.296), suggesting the dissociability of those two parameters in representing two dimensions of individual variability in Rawlsian preference. Both parameters showed good recoverability in the parameter recovery test, indicating they reliably contribute to accounting for cognitive processes underlying choice behavior in the task (Fig. S4, *α*: *r* = 0.98, *P*  *<* 0.001; φ: *r* = 0.92, *P* < 0.001).

### Neural sensitivity to the maximin computation predicts Rawlsian moral preference

As we found two distinct computations involved in Rawlsian preferences, we proceeded to examine their neural substrates. While our modeling results suggest that adherence to the maximin strategy and representation of agreeability are dissociable dimensions of Rawlsian preference at the algorithmic level, it is possible that processing the computation of two dimensions is subserved by either shared or distinct domains of neural processes at the level of implementation (40). If two dimensions engage common neural substrates, it may suggest two dimensions, while their algorithms differ, share the same underlying social motivation or goal at a higher level (41). On the other hand, if they engage distinct neural substrates, it would further suggest that they are psychologically distinct dimensions at both algorithmic and implementational level, composing individual variability of Rawlsian moral preference. With divergent characteristics of two dimensions in terms of directionality in stipulating Rawlsian preference, we adopted both univariate and multivariate approaches for neuroimaging analysis.

In terms of the maximin dimension, where more employment of the maximin strategy does scale with Rawlsian preference, we applied a univariate approach to identify brain regions of which the activity tracks decision value in favor of the Rawlsian option computed by the maximin strategy. Additionally, we tested whether individual variability in the maximin dimension of Rawlsian preference entails differential neural sensitivity to the difference in anticipated harm for an individual in the worst-off position. Based on the previous work highlighting the role of mentalizing in the motivation to protect the single individual (29, 30), we hypothesized encoding of maximin computation as well as individual differences in the degree to which one upholds this strategy is linked with brain activity in mentalizing network (dorsomedial prefrontal cortex, TPJ, and precuneus; 42–46).

**Figure 3:**
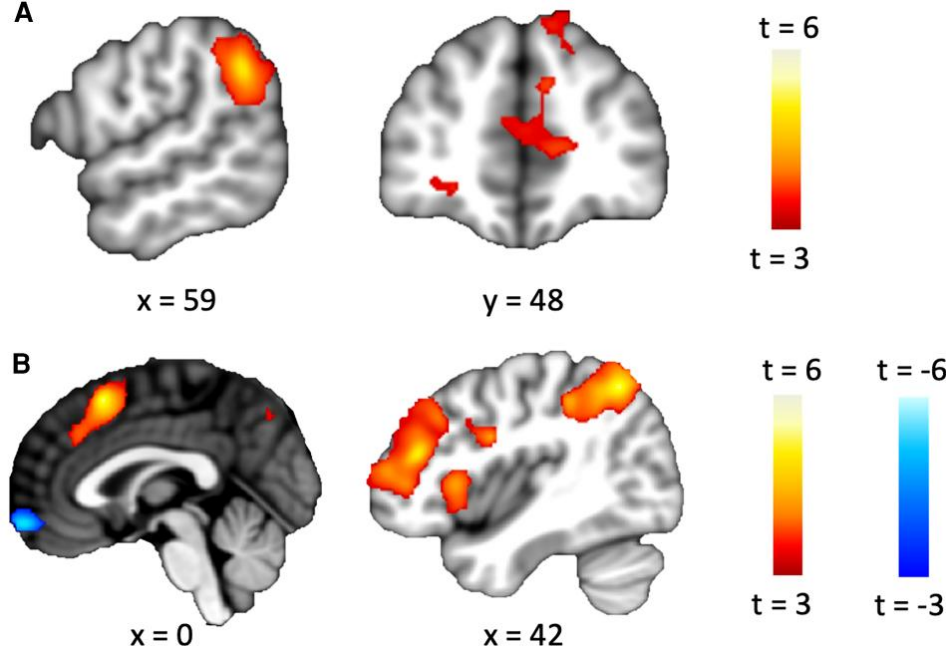
Rawlsian preference is associated with differential neural sensitivity to the maximin computation. A) The brain regions of the right TPJ/supramarginal gyrus, left middle frontal gyrus, and right medial frontal gyrus parametrically responded to the harm for one relative to an individual in the group $\left(C_{\mathrm{one}\;}>C_{\mathrm{group}\;}\right)$, which is the input to the maximin computation in Rawlsian strategy. B) Rawlsian moral preference is predicted by increased neural sensitivity in brain regions, including the bilateral insula, the inferior frontal gyrus, the inferior and superior parietal lobule, the right insula, and the left middle frontal gyrus. In addition, Rawlsian moral preference was associated with decreased neural sensitivity in vmPFC to the maximin computation. The whole-brain maps show brain regions where the parametric response to relative harm for the one versus the individual in the group correlated with the estimated parameter value of Rawlsian preference *α* from our model.

To first identify brain regions involved in maximin computation, we built a general linear model (GLM) that reveals parametric responses at choice onset, independently from participants’ choices, to the objective amounts of harm maximally assigned to an individual that would result from choosing the one relative to the group $\left(C_{\mathrm{one}\;}>C_{\mathrm{group}\;}\right)$. This essentially reflects the maximin computation of maximizing the minimum welfare (minimizing the maximum harm) imposed on an individual as predicted by the Rawlsian principle. A positive effect of this parametric contrast indicates the voxels encoding the extent of evidence in favor of the Rawlsian option arising from maximin computation. Consistent with our prediction, this contrast revealed an effect in the mentalizing network, including the right TPJ, left middle frontal gyrus, and right medial frontal gyrus, suggesting mentalizing is a key process involved in upholding the maximin strategy (Fig. 3).

Having found that the maximin computation is encoded in the activity of brain regions in the mentalizing network, we examined the hypothesis that varying neural sensitivity in those regions to evidence in favor of the Rawlsian option predicts individual differences in the maximin dimension of the Rawlsian moral preference. To this end, we added individual differences in moral preferences estimated from our winning model, parameter *α*, as second (group)-level regressor onto the parametric contrast of difference between the objective amounts of harm assigned to an individual in the one option compared with the group $\left(C_{\mathrm{one}\;}>C_{\mathrm{group}\;}\right)$. This analysis showed a positive link between the Rawlsian moral preference and neural responses to the maximin computation in the mentalizing network including the regions of the inferior frontal gyrus, superior and inferior parietal lobule, and superior frontal gyrus. The effect extended to other regions involved in social processing as well, including bilateral insula and left middle frontal gyrus. Interestingly, this analysis also revealed a negative link between Rawlsian preference and activity in regions implicated in valuation, including the ventromedial prefrontal cortex (vmPFC) and medial frontal gyrus, indicating neural responses to maximin computation in the valuation network scaled negatively with participants’ Rawlsian preference (Fig. 3). Functional connectivity analyses provided further suggestive evidence that individual differences in Rawlsian preference may be explained by the degree to which one incorporates concerns for the worst-off individual into integrative values of moral decision through mentalizing (see Supplementary Analysis S3).

### Neural signature of contractualist moral intuition

Next, we sought to examine the neural substrates of the “agreeability” dimension in Rawlsian preference. Importantly, unlike the maximin dimension where more employment of the maximin strategy does scale with Rawlsian preference, individual differences on the agreeability dimension are lacking such directional implication, as it is concerned with the representation of different ideas one considers agreeable to give more or less to an individual than the rest. Therefore, the univariate approach, which we applied to identify neural substrates of the maximin computation, would be less informative for addressing this question. Instead, we tested the hypothesis that participants who share similar ideas of agreeability are also likely to share brain responses to the encoding of harm ascribed to an individual. This reflects the idea of agreeability as the subjective threshold of what people deem as mutually agreeable, which we confirmed with our best computational model. We further predicted that varying deviations from such a threshold are likely to evoke neural activities that are similar among individuals who represent the equivalent or similar threshold.

To test this possibility, we employed the IS-RSA approach, which maps the representational distance across individuals in behavioral measures onto brain activity. By linking individual differences in the agreeability parameter we estimated from our model to brain data, it allowed us to identify neural substrates of the agreeability dimension of Rawlsian preference. Specifically, we examined which brain regions display shared brain patterns during the encoding of harm ascribed to the worst-off individual predicted by similarity in the agreeability parameter from our model. To this end, we ran dyadic regression models across voxels, where for every pair of participants in our data, neural similarity in encoding the amount of harm to the worst-off individual was regressed onto similarity in the agreeability parameter (φ). Critically, we controlled the similarity in the maximin parameter (α) in our regression model. This allowed us to examine similar neural responses to harm which is specifically attributed to the similarity in agreeability.

**Figure 4:**
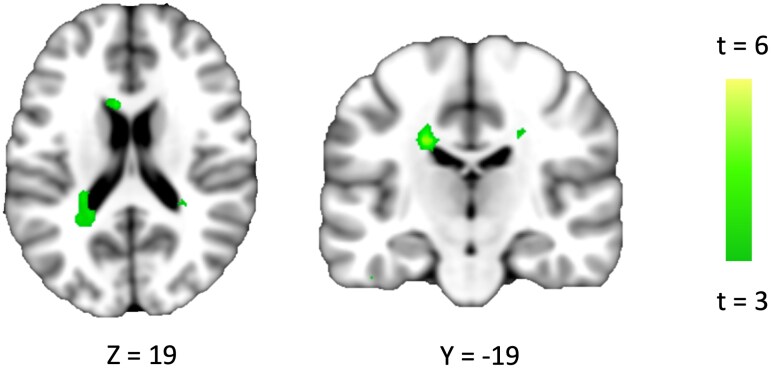
Similarity in the agreeability dimension predicts similar neural patterns encoding harm to the worst-off individual. Brain regions showing similar multivariate patterns encoding harm to the worst-off individual among participants who are similar on the Rawlsian agreeability dimension. Dyadic regression models were estimated at each voxel, in which neural similarity in encoding harm to the worst-off individual was regressed on similarity in model parameters. The beta estimates for the agreeability-dimension similarity were then mapped onto 3D brain space.

Our analysis revealed that the shared idea of agreeability predicted the extent to which clusters in the caudate and cingulate gyrus showed a similar response to the amount of harm that was disproportionately assigned to the worst-off individual. Specifically, activity in these regions showed that greater similarity in participants’ representations of agreeability—as indexed by the φ parameter—predicted greater similarity in their parametric neural responses to the amount of harm assigned to the worst-off individual (Fig. 4). The results are consistent with our computational model, where the maximin strategy and agreeability were represented by distinct parameters in computing decision value, but further suggest that those two dimensions are also implemented in distinct domains of neural processing, with the variance on the idea of agreeability uniquely mapped onto the neural encoding of harm on brain regions implicated in valuation.

## Discussion

In the current study, we investigated how people adjudicate conflicting normative moral principles when making decisions about allocations of harm. Our findings revealed distinct dimensions of the Rawlsian moral preferences in this context. We developed a new paradigm in which participants allocated harm between a single individual and a group of individuals. In most trials, minimizing the total amount of harm required delivering more harm to the one than to each member of the group, thus pitting Rawlsian moral principle of promoting fairness and justice among individuals against the utilitarian principle of maximizing aggregate outcome.

Participants in our task displayed strong Rawlsian preferences overall, predominantly choosing a less optimal option in terms of overall utility to ensure that the worst-off individual does not get a disproportionate amount of harm. This result extends the previous finding that people show a strong preference for the Rawlsian option in distribution decisions in the domain of fairness (47–49) and highlights how Rawlsian preference, as a distinct moral preference from Kantianism (rule-based deontology) and utilitarianism, manifests in weighing harm for individuals versus groups.

Kantian moral preference, which makes the prediction that people will distinguish “action” from inaction, was not supported in the context of our task, with the Rawlsian preference overruling its impact for participants’ choices. Influential work investigating people's responses to sacrificial moral dilemmas showed the dominance of Kantian judgments in a scenario where it involved direct and personal harm as an instrumental means to save a greater number of people (2, 8, 50, 51). Moral dilemmas used in previous research are usually designed in a way that causes harm to the most disadvantaged individuals in a way that involves action of direct and personal harm. As a result, previous studies indicating a Kantian preference may actually be indicative of a Rawlsian preference. The methods employed in the past did not allow for the distinction between these two preferences. Therefore, many of the findings that have highlighted the prevalence of Kantian preference could be interpreted as reflecting a Rawlsian preference. While Rawlsian and Kantian principles both fall under the umbrella of deontology, our work suggests that their underlying psychological profiles are distinguishable from each other.

Most participants in our study showed a moral preference that is a mixture between Rawlsian and utilitarian principles, while more heavily weighted toward the Rawlsian one. The trade-off between the amount of harm allocated to an individual and group revealed the extent to which people are aligned with the two competing moral principles. Specifically, increasing the maximum amount of harm for the worst-off individual led to favoring the Rawlsian option over the utilitarian option, while increasing the number of harm recipients or the overall amount of harm for the group led to favoring the utilitarian option. Participants’ moral decisions were best explained by our computational model, which quantified the subjective value of choices by decomposing distinct dimensions of individual variability in Rawlsian moral preferences.

Past research on the psychological profile of Rawlsian moral preferences has mainly focused on the maximin Rawlsian strategy in its competition with the utilitarian strategy (30). Our computational model also confirmed the maximin Rawlsian strategy in the context of harm allocation, demonstrating the motivation to protect an individual from the worst-off outcome at the cost of increasing overall harm (and therefore maximizing the minimum welfare). However, we further illuminated another dimension of Rawlsian moral preference that is distinct from the employment of maximin strategy. Theoretically motivated by the contractualist basis of Rawls's theory of justice (15), we hypothesized that people would represent different ideas of agreeability grounded in the mutual respect of individuals’ perspectives. Incorporating this agreeability dimension to our computational model identified an important source of individual variability in Rawlsian moral preferences. Capturing different subjective thresholds of what is deemed an acceptable amount of harm to be imposed on one person, it significantly improved the model fit to the behavioral data.

Importantly, the individual variability in maximin and agreeability dimensions of Rawlsian moral preference was not significantly correlated, suggesting those two dimensions play distinct roles in explaining individual variability of Rawlsian preference. We propose that this contractualist intuition, while not directly pitted against the utilitarian strategy of maximizing the aggregate outcome, offers a distinct motivation in the endorsement of the Rawlsian principle. In the context of harm allocation, the agreeability dimension reflects consideration of what is a reasonable amount of harm that an affected individual would agree to receive more or less than the rest. By representing the standpoint of an individual in an impartial manner, it is more likely that the resulting decision will distribute burdens in a way that is more fair and justifiable even to the worst-off. Therefore, the agreeability dimension can lead to protecting the mutual respect and balance between individuals’ rights so that no one gets disproportionately burdened or sacrificed for enhancing aggregate outcomes.

Our work highlights psychological profiles of Rawlsian preference that are shared across harm and fairness domains. We found that computation of the Rawlsian maximin strategy as well as its individual variability was associated with the activity of the mentalizing network in the brain. This is consistent with previous work that investigated the neural basis of the maximin strategy in the domain of fairness and risky decision-making (30). This also dovetails with the observation that mPFC is more engaged in response to events of a single person than a group of individuals, demonstrating the neural underpinnings of the singularity effect (29). Therefore, we provide a unified explanation as to why people may dislike an outcome that does not share the greater good with the worst-off, regardless of whether the allocated outcome is less benefit or more burdens. Our tendency to help the worst-off despite the cost of reduced utility is driven by taking the perspective of the worst-off and inferring what it is like to be situated in that particular position for getting to experience unjust distribution of benefits and burdens.

Finally, we investigated the neural profile of the agreeability dimension of Rawlsian preference by applying a multivariate approach. With the IS-RSA approach that allowed mapping between interindividual variation in model parameter and brain activity, we examined similar neural engagement in parametric response to the worst-off harm predicted by similarity in the idea of agreeability. We found that the caudate and cingulate gyrus showed similar responses to the amount of harm that was assigned to the worst-off individual. Our finding that two dimensions of Rawlsian preference can be respectively mapped onto distinct neural regions suggests that they are dissociable both at the level of computation and implementation. Moreover, given the previous work suggesting the role of the caudate in tracking the integration of equity and utility (52), the neural profile of the agreeability dimension we identified here seems to reflect the evaluation of the worst-off individual's position from an impartial standpoint. We speculate that the caudate plays a key role in maintaining reasonable balance in individuals’ share of the greater good by tracking how much a share for the worst-off position deviates from one's internal threshold of agreeability.

We acknowledge that practical constraints, including available funding and recruitment limitations during the COVID-19 pandemic, constrained the final sample size. As a result, the sample size was not determined in advance, which may limit the strength of our inferences. Replication with larger samples will be important to evaluate the robustness and generalizability of the effects reported here. In addition, because our data were collected in South Korea, we caution against making broad general claims about how concern for harming an individual reflects overall moral preferences, as moral values and judgment patterns can vary across cultures (53, 54). Rather, we view our primary contribution as demonstrating that concern for harming an individual comprises psychologically distinct dimensions. We hope that the novel task we developed here to dissociate deontological moral preferences will facilitate future work examining this computational dissociation and testing whether and how these processes manifest similarly or differently across cultures.

To conclude, our study elucidates the neurocomputational process involved in harm allocation decisions, adjudicating normative moral principles beyond the conflict between categorical deontic prescriptions and maximizing aggregate welfare. Our results demonstrate that Rawlsian moral preference, as separate from Kantian deontological preference, can be further decomposed into two distinct neurocognitive aspects. Our findings underscore the significance of broader deontological principles and highlight the nuanced nature of individuals’ moral preferences within normative ethical theories. Formalizing the psychological mechanisms of our moral preferences in their diverse dimensions contributes to a more comprehensive understanding of the complex landscape of human morality, enriching our knowledge of how they shape and guide our social behaviors.

## Methods

### Participants

Sixty-eight participants were recruited via a university community website as paid volunteers. Exclusion criteria were history of neurological disorders, psychiatric disorders, use of psychoactive medication or drugs, and pregnancy or suspicion of pregnancy. Participants who majored in psychology or who had previously participated in social psychology studies were also excluded due to concerns that previous experience with psychology studies involving deception could increase the suspicion about the task in the current study. All participants were right-handed, had normal or corrected-to-normal vision, and fulfilled safety criteria for cold-pressor tests and MRIs. Sixteen participants were excluded from the fMRI analysis because six participants attended only the behavioral parts of the study due to the technical issues, four participants fell asleep in the scanner, one participant requested to exit the scanner during the first run, one participant had focal brain atrophy that was not identified prior to the scanning, and four participants had excessive head movement (>3 mm) during the scanning, leaving a total of 52 participants whose data were analyzed for the fMRI study (18 females, 34 males; mean age 23.27 ± 3.06). We reported the behavioral results from analyzing the data of all 68 participants who completed the behavioral task (29 females, 39 males; mean age 23.43 ± 3.21).

### Procedure

The procedure of the study was approved by the Institutional Review Board at Seoul National University (1907/003-007). The study consisted of a 2-h-long session. At the start of the study, the experimenter explained the procedure and the purpose of the study. To make participants believe their decisions will be actually implemented and have real consequences for other individuals, we informed participants that the aim of the current experiment was 2-fold: (i) to examine people's preferences in making social decisions and (ii) to decide parameters for a “group-decision-making” task in another study of the lab.

Specifically, at the beginning of the experiment, participants were told that one randomly selected trial from today's task could be implemented in a related study involving other participants. They were informed that the related study examines exposure to a discriminatory–stress scenario and its effects on cooperative decision-making in a separate group task, and to ensure the manipulation involved an appropriate level of differential stress, we would use the option selected from the participant's responses. Thus, the group-decision-making task (which was not implemented in reality) served as a cover story to justify the presence of other participants who would purportedly receive different levels of the stress manipulation as a consequence of the participant's decision during the task. To increase the credibility of the cover story, we asked participants to sign the informed consent for using the participant's decision as well as for waiving future participation in the study of the group-decision-making task.

After providing the informed consent, a protocol for saliva collection was administered for data to be reported for the purpose of a different study. Participants then completed a battery of trait questionnaires, which was followed by the instruction about the harm allocation task. Participants were told that at the end of the task, one trial would be randomly selected, and their decision in that trial would be implemented for a group of participants in another study in the lab. We emphasized that there are no right or wrong answers, and participants shall make choices based on their own preference. In order to minimize the concerns about reputation or reciprocity and their impact on participants’ choices, we informed participants that their choices and identity would be kept confidential to participants in another study of the lab getting affected by the participant's decision.

Before the scanning, participants first completed a 20-s cold-pressor test at 4 °C water. We specifically chose to administer a cold-pressor test for 20 s given it is not long enough to develop numbness with respect to the increase of the time. Also, this was shorter than any pair of options presented in the task, allowing us to examine how people make decisions that have impact on other individuals to some extent beyond their own experience. This procedure allowed participants to experience pain involved in a cold-pressor test before the harm allocation task, where they were supposed to believe that their decision of allocating the amount of harm as time of getting this test involving pain will be actually implemented to other individuals. Following the administration of the cold-pressor test, the participant completed the harm allocation task in the fMRI scanner.

After completing the scanning, participants completed a short debriefing questionnaire asking about their experience and beliefs about the experimental setup, including a question of how much they believed that their choices would be actually implemented to other individuals (rated from 1 = “not at all” to 5 = “fully”). Participants overall reported high levels of belief that the cover story of our study would be real (*M* = 3.96, SEM = 0.16). Participants also reported high confidence that their choices and identity would remain confidential (choices: *M* = 4.80, SEM = 0.07, identity: *M* = 4.77, SEM = 0.07), suggesting concerns about reputation or reciprocity had little or no influence over participants’ choices in the task (see Supplementary Analysis S2 for a further manipulation check examining whether participants’ beliefs about the procedure influenced task behavior). All the participants were fully debriefed about the deception before departing the laboratory.

### Harm allocation task

On every trial, participants had to choose between allocating harm to a single individual (the one) and a group of individuals (the group). The one option contained more harm (longer time of getting a cold-pressor test) in terms of what was maximally given to an individual, while the group option contained more harm in total. Specifically, the amount of harm in the one option $\left(C_{\mathrm{one}}\right)$ was always the same or bigger than the amount of harm allocated to each individual in the group option $\left(C_{\mathrm{group}}\right)$ but was always the same or smaller than the total amount of time involved in the group option $\left(C_{\mathrm{group}}\times N_{\mathrm{group}}\right)$, thus satisfying the following property: $C_{\mathrm{group}}\leq C_{\mathrm{one}}\leq C_{\mathrm{group}}\times N_{\mathrm{group}}$. This allowed us to examine how participants pit the maximin Rawlsian strategy against the utilitarian strategy of maximizing the overall amount of welfare (or minimizing the overall amount of harm). Here, Rawlsian strategy indicates maximizing the welfare of the worst-off position of getting the most amount of harm, so choosing the group to minimize the maximum harm at the cost of imposing more harm overall. On the other hand, the utilitarian strategy concerns bringing the better outcome in the aggregated level and, therefore, choosing the one to minimize the overall amount of harm.

In both the one option and the group option, the amount of time ranged from 30 to 180 s in increments of 10, with the number of individuals in the group option alternating between three and four. The 50 choice pairs satisfying the aforementioned conditions were predetermined and repeated across three conditions, which differed in the default option that was implicated when participants were not pressing any button. The default option was indicated by the direction of the lever in the middle of the screen. Varying the default option across the same set of trials allowed us to examine the participant's preference for the default option as predicted by Kantian theory. Duplicating the choice pairs three times resulted in 150 trials (50 trial pairs × 3 conditions) evenly distributed and delivered across three scanning runs lasting ∼8 min each. We randomized the presentation order of the trial as well as the location of the one and the group options for every trial.

After 3 s of the wait phase, where the choice pair and the default condition are revealed, participants had a maximum of 2.5 s to choose either option by pressing a button box with their right index or middle finger. Button presses in the selection of an alternative option resulted in shifting the lever, directing the ball to move toward the chosen option and hitting the box surrounding the option in 0.5 s. The selected option was highlighted for 1.5 s. The end of each trial was followed by a jittered fixation cross (0.5–2 s).

### Computational modeling and model comparison

Model fitting and comparison were performed using hierarchical Bayesian estimation (36–38) using the package RStan (Stan Development Team 2020) in R (R Core Team 2020). The individual parameters were treated as a random sample drawn from a group distribution, and individual and group parameters were estimated simultaneously in a mutually constraining manner. We employed weakly informative priors for group-level parameters and the noncentered parameterization (Matt trick) method for optimizing the sampling process (55). The posterior distribution of parameters was simulated by means of four independent Markov Chain Monte Carlo (MCMCs) sampling chains. Each chain consisted of 1,000 MCMC samples after discarding 1,000 warm-up samples, for a total of 4,000 posterior samples. We compared the goodness of fit of different models using LOOIC as a metric (39). The model with the lowest LOOIC score was selected as our winning model and used for subsequent analysis. Posterior predictive checks were carried out by simulating choice data based on the parameters derived from the winning model.

The weighting parameter *α* indicated how much participants employed the Rawlsian strategy or the utilitarian strategy. It can take any value between 0 and 1, and when *α* = 0, it corresponds to pure utilitarian preferences and calculates the difference in utility between two options solely based on the difference in the total amount of harm, and therefore always ends up choosing the one option that has lower overall harm. On the other hand, when *α* = 1, it takes the maximin Rawlsian strategy, and the subjective utility of two options will rely on the difference between the amounts of harm that each individual gets and thus going for the group option that maximizes the minimum welfare for an individual. We compared the model that allowed this parameter to freely have a value between 0 and 1 to the models where the weighting parameter is constrained to either 0 or 1 and tested whether people balanced the maximin and utilitarian strategies or relied on only one of them in harm allocation decisions.

Next, we considered the agreeability parameter (*φ*), which we assumed to be a separate dimension of Rawlsian moral preference. It is possible that people represent the threshold of agreeability either as the ratio or as the difference between the amounts of harm that individuals get. Therefore, we tested which type of agreeability parameter (constant or ratio term) leads to better fit to the data. If the model with a ratio term excels in model fit, it would suggest that people represent the agreeable amount of differential harm between individuals in a multiplicative manner (e.g. people think it is reasonable for the worst-off individual to receive twice as much harm as the rest). On the other hand, if the model with a constant term excels, it would indicate that people represent the agreeable amount of differential harm between individuals either as a decremental or as an incremental amount.

Our model comparison result indicated that the model that explained our choice data best was a model with three free parameters, which posits the difference in subjective utility between the Rawlsian option and the utilitarian option is as follows:


$$U(\mathrm{one})=\;-(1-\alpha )\;C_{\mathrm{one}}-\alpha \;(\;C_{\mathrm{one}}+\varphi \;)\;$$

$$U(\mathrm{group})=\;-(1-\alpha )\;C_{\mathrm{group}}\;N_{\mathrm{group}}-\alpha \;C_{\mathrm{group}}$$

ΔU=(1−α)(Δtotal)−α(Δsingle+φ)

$$\;\Delta \;\mathrm{total}\;\;=\;C_{\mathrm{group}\;}N_{\mathrm{group}}-C_{\mathrm{one}}$$

$$\;\Delta \;\mathrm{single}\;\;=\;C_{\mathrm{one}\;}-C_{\mathrm{group}}$$

Here, ΔU denotes the subjective utility of choosing the Rawlsian option over the utilitarian option which was determined by the two parameters, *α* and *φ*, capturing different aspects of Rawlsian moral preferences. The weighting parameter that freely varied between 0 and 1 was able to capture how much participants preferred the Rawlsian strategy over the utilitarian strategy, indicating most of the participants preferred a mixture between the two. Additionally, the best model was with the agreeability parameter with a constant term, suggesting that the agreeable amount of differential harm between individuals is represented either as a decremental or as an incremental amount rather than a ratio.

After the utility comparison between the two options was computed based on those two free parameters, the softmax function was applied to calculate the choice probabilities. For the softmax function, we considered an inverse temperature parameter, *τ* (tau), to take into account stochasticity in value-based choices.


$$P\;(\mathrm{choose}\;\mathrm{one})=\frac{1}{1+e^{-\tau \;\Delta U}}$$

The winning model was able to accurately reproduce the observed proportion of Rawlsian decisions (*R* = 0.99, *P* < 0.001). We also carried out the parameter recovery test to check the identifiability of model parameters (55). This was done by comparing the estimated parameter values on simulated data with “true” parameters used to generate the data with our winning model. Based on the winning model and its parameter estimation, the mean of the chosen and unchosen options’ subjective utility was calculated in a trial-by-trial manner and used as regressors of parametric modulation in GLM2.

### fMRI acquisition and preprocessing

fMRI scanning was performed on a 3T Siemens Magnetom Trio MRI scanner at Seoul National University, South Korea. Functional images were obtained using a multiband $T_{2}^{*}$-weighted echo-planar imaging sequence using the following parameters: repetition time = 1.5, echo time = 30 ms, flip angle = 85°, 64 slices, field of view = 256 mm, voxel size = 2.3 × 2.3 × 2.3 mm, slice thickness = 2.3 mm. We acquired 295 volumes in each of three sessions. T_1_-weighted anatomical images were acquired with magnetization-prepared rapid gradient echo sequence with the following parameters: repetition time = 2.3, echo time = 2.36 ms, field of view = 256 mm, voxel size = 1 × 1 × 1 mm, slice thickness = 1 mm.

Preprocessing of fMRI data was performed using fMRIPrep 1.4.0, which is based on “Nipype” 1.2.0 (56, 57; RRID:SCR_002502). Preprocessed images were spatially smoothed with a Gaussian kernel of full width at half maximum = 8 mm and analyzed using SPM12.

### GLM1: model of maximin computation

The first GLM was built to determine brain regions showing parametric responses at decision onset, independently from participants’ choices, to the objective amount of harm that would be assigned to an individual at maximum from choosing the one relative to the group, as indicated by the Rawlsian maximin strategy. fMRI time series were regressed onto a GLM with the onset of the decision phase as the main event regressor. The event was modeled with a duration corresponding to the participant's RT on that trial. Two parametric modulators were added for each regressor of onset: the objective amount of harm (the amount of time) involved in the one option and in the group option for each individual. The GLM contained five additional event regressors of no interest, describing the onsets of (i) the wait phase, (ii) implementing phase 1 (selected option being implemented), (iii) implementing phase 2 (selected option being highlighted), (iv) the fixation cross, and (v) button presses. The event of each phase in the task was modeled with its corresponding duration, while the event of the button press was modeled as a stick function (duration = 0 s). We included a series of nuisance regressors of no interest to control for motion effects. Specifically, we added six motion regressors obtained during realignment and spike regressors determined on the basis of FD and DVARS thresholds within the fMRIPrep pipeline. The number of spike regressors varied by individual depending on the degree of their head motion during the scanning.

### GLM2: model of relative chosen value

We constructed the second GLM to determine brain regions encoding the utility of chosen and unchosen options at decision onset. The utility of options was identified by our winning model. The main event regressor was the onset of the decision phase, which was modeled with a duration corresponding to the participant's RT on that trial. Two parametric modulators were entered for each regressor of onset: the subjective value of the chosen and unchosen options, derived from each participant's choice model. We added the same set of event regressors and nuisance regressors of no interest as in GLM1. All univariate fMRI results reported in the results survive whole-brain correction for multiple comparisons (*P* < 0.05, FWE corrected at the cluster level following voxel-wise thresholding at *P* < 0.001)

### IS-RSA approach

To probe whether participants who share a similar idea of agreeability also show similar brain responses in encoding harm during moral decision-making, we applied the IS-RSA approach and examined the association between similarity in the agreeability parameter and the similar neural responses. The analysis was performed with dyadic regression models implemented on R code adapted from van Baar et al. (58). The main aim of running the dyadic regression model was to identify brain regions where similarity in agreeability parameter predicted similar neural engagement (58, 59). We examined the similar neural engagement in the parametric response to harm assigned to individuals in the worst-off position. We controlled for the similarity in the maximin parameter to ensure the effect is specific to similarity in agreeability.

To this end, first, we created a geometric representation of individual variabilities in variables of our interest: each dimension of Rawlsian preference (separately for the agreeability dimension and the maximin dimension) and neural similarity in the parametric BOLD response to the worst-off harm amount for each voxel we obtained from the aforementioned GLM. The geometric representations for those variables were obtained by computing similarity between all pairs of participants calculated as the Euclidean distance from the observation of two participants converted to a similarity score range between 0 and 1 using the following formula:


$$\mathrm{Similarity}\;\mathrm{score}=\frac{1}{1+d(S_{1},\;S_{2})},$$

where *d*(*S*_1_, *S*_2_) is the Euclidean distance between two subjects *S*_1_ and *S*_2_.

This procedure allowed us to have all our variables of interest expressed on the same range between 0 and 1.

The dyadic regression models mapped the geometric representation of behavioral data to brain data by regressing similarity in beta estimates onto similarity in the agreeability parameter with covariates of similarity in the maximin parameter. This allowed us to identify similar multivariate neural patterns that were specifically associated with similarity in agreeability parameter while controlling for similarity in the maximin strategy. Random intercepts of participants were included in our dyadic regression model to correct for statistical dependencies that arise from the repeated occurrence of individual data in pairwise observation. The dyadic regression model was estimated for each individual voxel, where the observations corresponded to all possible pairs of participants without repetition. The beta estimates of the agreeability dimension from dyadic regression models were mapped onto 3D brain space to generate the resulting beta map. We tested for statistical significance of the beta map using voxel-wise thresholding at *P* (FDR) < 0.05 and reported surviving clusters with the size of five or more contiguous voxels, following the same threshold used in a recent work that employed the same approach (58).

## Supplementary Material

pgag074_Supplementary_Data

## Data Availability

Behavioral data, analysis scripts, computational modeling, task code, and unthresholded statistical maps of fMRI data have been deposited in the OSF repository and are publicly available at https://osf.io/2huxq/ (60) (https://doi.org/10.17605/osf.io/2huxq).
