# Supplementary material for: Decomposing the neurocomputational mechanisms of deontological moral preferences
Source: PNAS Nexus. 2026 Apr 7;5(4):pgag074. doi: 10.1093/pnasnexus/pgag074 (PMC13064635; doi:10.1093/pnasnexus/pgag074)
Supplement: pgag074_Supplementary_Data [file pgag074_supplementary_data.docx]

**Supplementary Materials**

**Supplementary Table 1 :** Computational Model Comparisons

| **Model** | **Parameters** | **LOOIC** |
| --- | --- | --- |
| Model 1  (Best Model) | 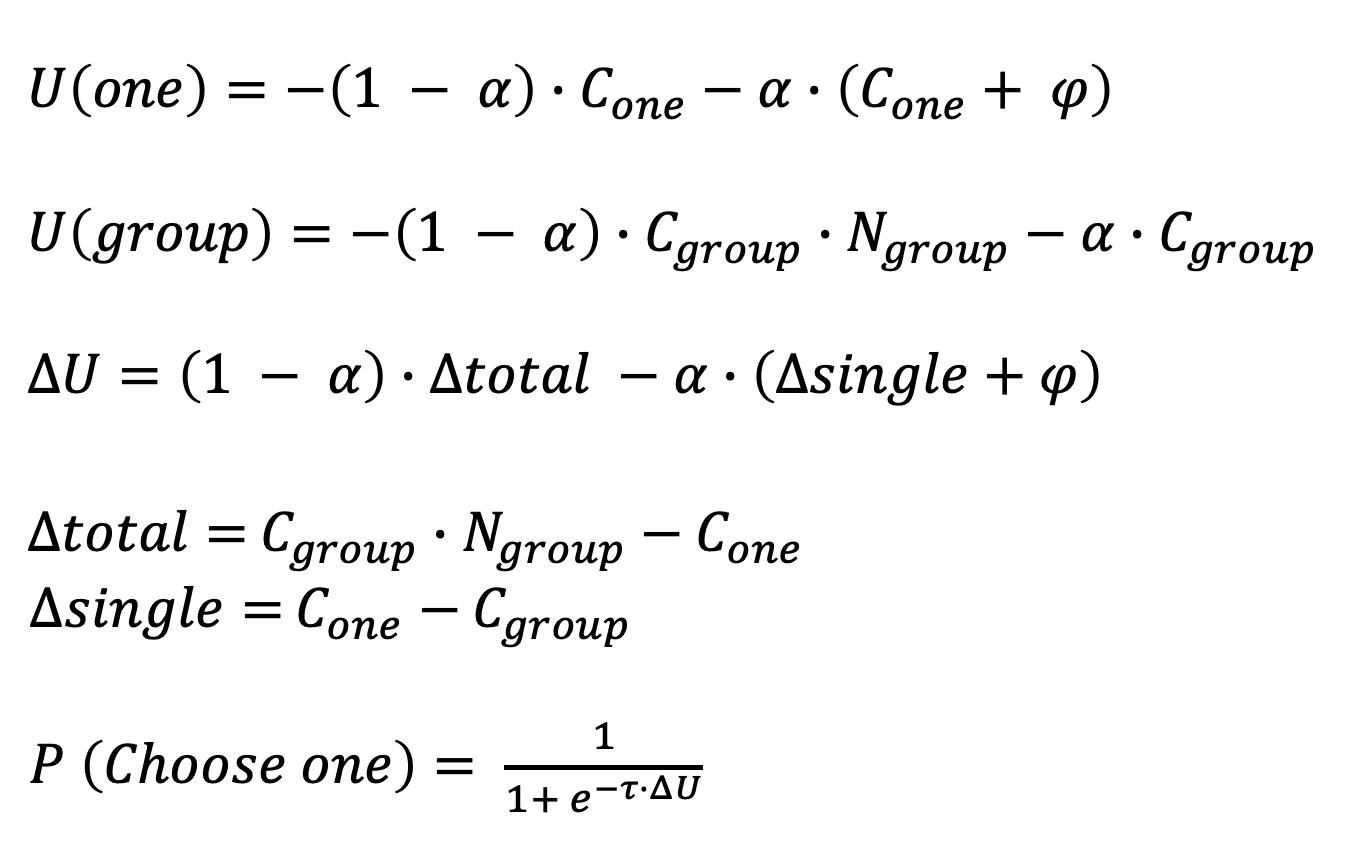 | 6112.0 |
| Model 2  (alpha constrained to 0) | 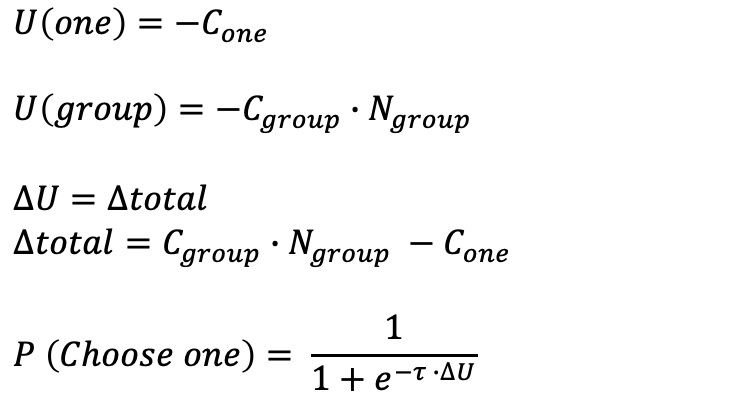 | 12989.0 |
| Model 3  (alpha constrained to 1) | 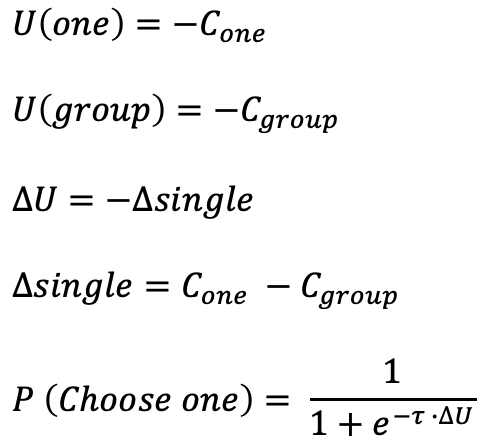 | 10400.4 |
| Model 4  (no agreeability parameter) | 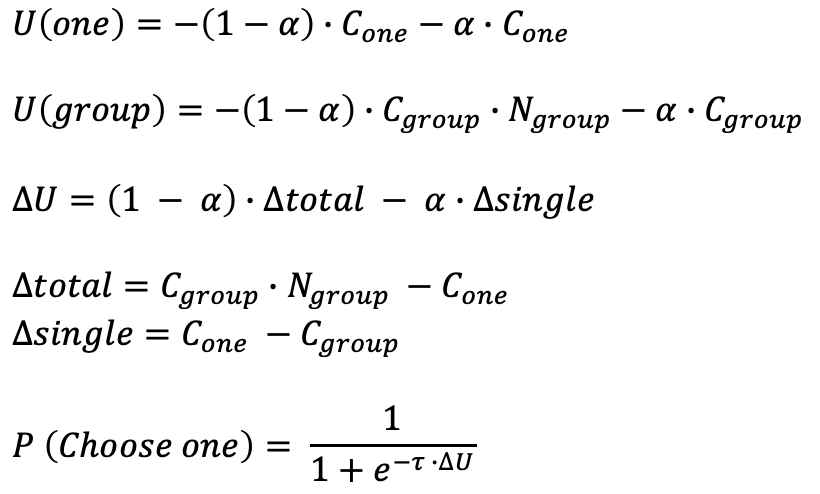 | 6653.5 |
| Model 5  (agreeability parameter as multiplicative term) | 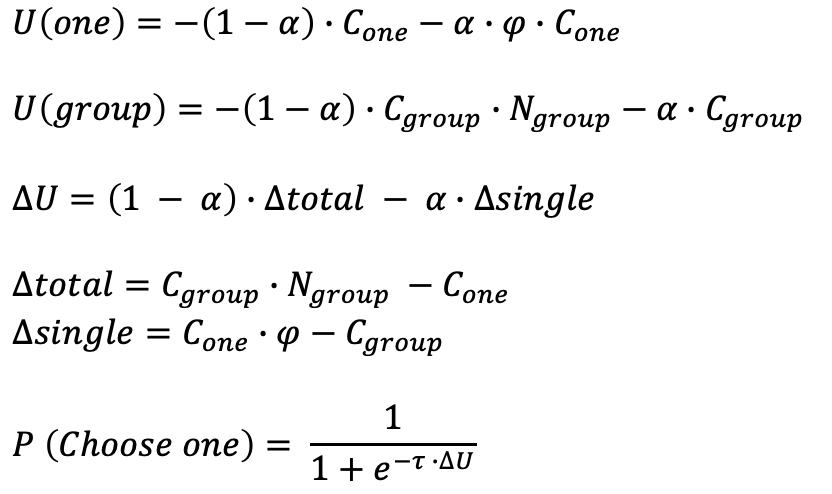 | 6463.4 |

**Supplementary Table 2 :** GLM1 : Cone > Cgroup

| **Region Name** | **Extent** | **t-value** | **x** | **y** | **z** |
| --- | --- | --- | --- | --- | --- |
| Supra Marginal Gyrus / rTPJ | 678 | 5.50 | 66 | -49 | 30 |
| Mid Frontal Gyrus | 154 | 4.52 | -34 | 37 | 41 |
|  |  | 3.53 | -27 | 49 | 43 |
| Inf Frontal Gyrus | 252 | 4.30 | -31 | 40 | -12 |
|  |  | 3.57 | -24 | 49 | -10 |
|  |  | 3.44 | -29 | 68 | -7 |
| Mid Temporal Gyrus | 173 | 4.14 | 62 | -18 | -12 |
| Superior Frontal Gyrus | 136 | 3.93 | 20 | 28 | 64 |
|  |  | 3.73 | 22 | 19 | 69 |
| dmPFC | 392 | 3.84 | 8 | 47 | 27 |
|  |  | 3.82 | 11 | 49 | 4 |
|  |  | 3.57 | 15 | 40 | 32 |
| vlPFC | 172 | 3.76 | 48 | 37 | -16 |
|  |  | 3.55 | 52 | 47 | -14 |
| Superior Frontal Gyrus/ dlPFC | 53 | 3.64 | 31 | 40 | 48 |

**Supplementary Table 3 :** GLM1, multiple regression : Cone > Cgroup; positive correlation with alpha

| **Region Name** | **Extent** | **t-value** | **x** | **y** | **z** |
| --- | --- | --- | --- | --- | --- |
| Inferior Frontal Gyrus / dlPFC | 2720 | 7.88 | 57 | 12 | 27 |
|  |  | 6.42 | 45 | 2 | 27 |
|  |  | 5.79 | 41 | 37 | 20 |
| Superior Parietal Lobe/ Precuneus | 2577 | 7.10 | 31 | -60 | 48 |
|  |  | 6.86 | 50 | -39 | 57 |
|  |  | 6.11 | 29 | -49 | 41 |
| dmPFC / Superior Frontal Gyrus | 1380 | 6.29 | 1 | 19 | 55 |
|  |  | 5.85 | 11 | 26 | 34 |
|  |  | 5.58 | -8 | 12 | 53 |
| lTPJ / Inferior Parietal Lobule | 1638 | 6.25 | -45 | -42 | 48 |
|  |  | 5.66 | -27 | -72 | 55 |
|  |  | 5.55 | -22 | -60 | 48 |
| Insula | 556 | 5.84 | 34 | 19 | 9 |
|  |  | 5.77 | 34 | 23 | -5 |
|  |  | 5.30 | 45 | 16 | 7 |
| Mid Temporal Gyrus | 247 | 5.44 | 55 | -39 | -16 |
|  |  | 5.02 | 50 | -53 | -12 |
| Insula | 1212 | 5.13 | -31 | 28 | 7 |
|  |  | 5.00 | -52 | 35 | 25 |
|  |  | 4.90 | -48 | 7 | 32 |
| Fusiform Gyrus | 386 | 4.75 | -38 | -74 | -7 |
|  |  | 4.31 | -34 | -65 | -28 |
| Mid Frontal Gyrus | 348 | 4.37 | -22 | -5 | 57 |
|  |  | 4.11 | -27 | -14 | 50 |
|  |  | 3.90 | -38 | 0 | 55 |

**Supplementary Table 4 :** GLM1, multiple regression: Cone > Cgroup ; negative correlation with alpha

| **Region Name** | **Extent** | **t-value** | **x** | **y** | **z** |
| --- | --- | --- | --- | --- | --- |
| vmPFC | 288 | 5.59 | -1 | 61 | -12 |
|  |  | 4.47 | 11 | 51 | -14 |
|  |  | 3.61 | 6 | 37 | -12 |
| Inferior Frontal Gyrus / vlPFC | 204 | 4.90 | 41 | 9 | -21 |
|  |  | 4.79 | 52 | -7 | -21 |

**Supplementary Table 5 :** GLM2, Chosen utility > Unchosen utility

| **Region Name** | **Extent** | **t-value** | **x** | **y** | **z** |
| --- | --- | --- | --- | --- | --- |
| Posterior Cingulate Cortex | 3744 | 7.27 | 20 | -46 | 13 |
|  |  | 6.66 | -10 | -56 | 16 |
|  |  | 6.61 | 25 | -42 | 20 |
| Caudate | 1552 | 7.07 | 6 | 16 | -7 |
|  |  | 6.52 | 4 | 26 | -7 |
|  |  | 6.15 | 6 | 37 | -5 |
| Mid Temporal Gyrus | 226 | 6.23 | -45 | -77 | 30 |
|  |  | 3.61 | -38 | -63 | 27 |
| Mid Temporal Gyrus | 316 | 5.62 | -59 | -5 | -14 |

**Supplementary Table 6:** IS-RSA analysis result showing similar multivariate patterns of neural encoding of harm to worst-off individual in participants with similar value of agreement parameter

| **Region Name** | **Extent** | **t-value** | **x** | **y** | **z** |
| --- | --- | --- | --- | --- | --- |
| Cingulate Gyrus | 144 | 5.76 | -20 | -40 | 30 |
| Caudate | 72 | 5.41 | -22 | -42 | 10 |
| Caudate | 176 | 5.38 | -20 | -20 | 32 |
| Cerebellar Tonsil | 64 | 5.09 | -10 | -44 | -60 |

**Supplementary Table 7 :** PPI Choose Rawlsian > Choose Utilitarian ; positive correlation with alpha

| **Region Name** | **Extent** | **t-value** | **x** | **y** | **z** |
| --- | --- | --- | --- | --- | --- |
| dmPFC | 292 | 5.27 | 4 | 54 | 16 |
|  |  | 5.08 | 4 | 54 | 34 |
|  |  | 4.91 | 6 | 63 | 20 |

**Supplementary Table 8:** PPI result for contrast of Choose Rawlsian > Choose Utilitarian ; negative correlation with alpha

| **Region Name** | **Extent** | **t-value** | **x** | **y** | **z** |
| --- | --- | --- | --- | --- | --- |
| Fusiform Gyrus | 27962 | 7.54 | 48 | -44 | -14 |
|  |  | 7.35 | -15 | 12 | 30 |
|  |  | 7.27 | 13 | -53 | 0 |
| Cingulate Gyrus | 254 | 5.41 | -13 | -23 | 43 |
|  |  | 5.20 | 11 | -37 | 39 |
|  |  | 5.18 | -8 | -32 | 39 |
| Anterior Cingulate Cortex | 154 | 4.62 | -13 | 42 | 0 |
|  |  | 4.26 | -3 | 54 | -3 |

**Supplementary Figure 1.** Example trials shown to participants during the task.


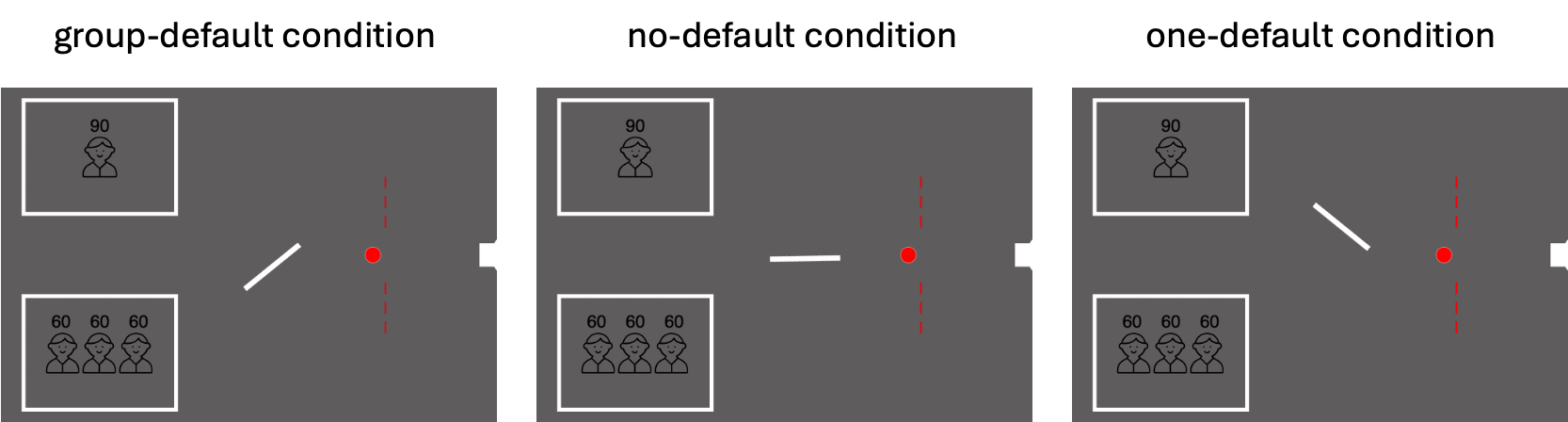


Example trial shown to participants under three default conditions. During the choice stage, when the ball crosses the dotted line, participants can press a button to switch the lever and redirect the ball toward the other option. In the group-default condition, the lever is initially set toward the group option, such that the ball rolls toward the group option unless participants switch (i.e., the group option is the default). In the no-default condition, the lever is not pre-set, and participants must actively choose between the two options. In the one-default condition, the lever is initially set toward the one-person option, such that the ball rolls toward the one-person option unless participants switch to the group option.

**Supplementary Figure 2**.The correlation between actual choice ratio and model predicted choice


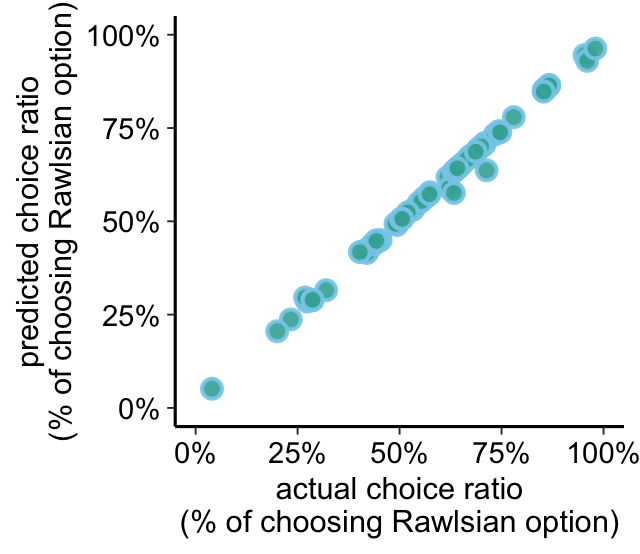


The best-fitting model we identified through model comparison accurately predicted choice behavior in our task, with a strong correlation between the model’s prediction and observed choice ratio of Rawlsian option (r = 0.99, p < .001).

**Supplementary Figure 3.**Relationships between α and φ parameters, and between each parameter and Rawlsian choice preference

**
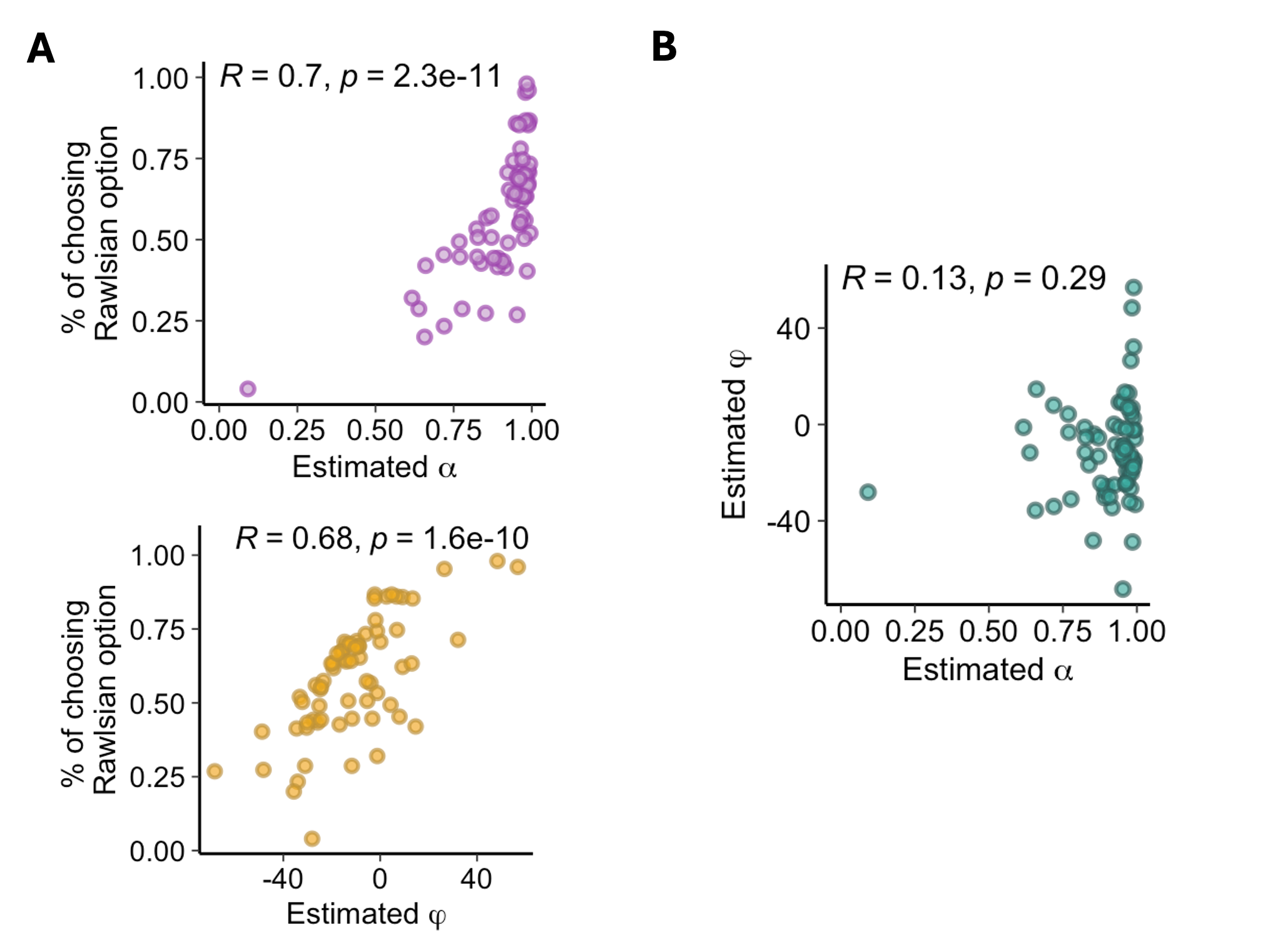
**

Both α and φ parameters were associated with higher rate of choosing Rawlsian option (panel A: α: *r* = 0.70 , *p* <.001 , φ: *r* = 0.68, *p* <.001). However, the two parameters were only weakly correlated with each other and this association was not statistically significant (panel B: *r* = 0.13, *p* = 0.289). This pattern supports a conceptual distinction between the two computations captured by the model.

**Supplementary Figure 4.** Parameter Recovery for three model parameters

**
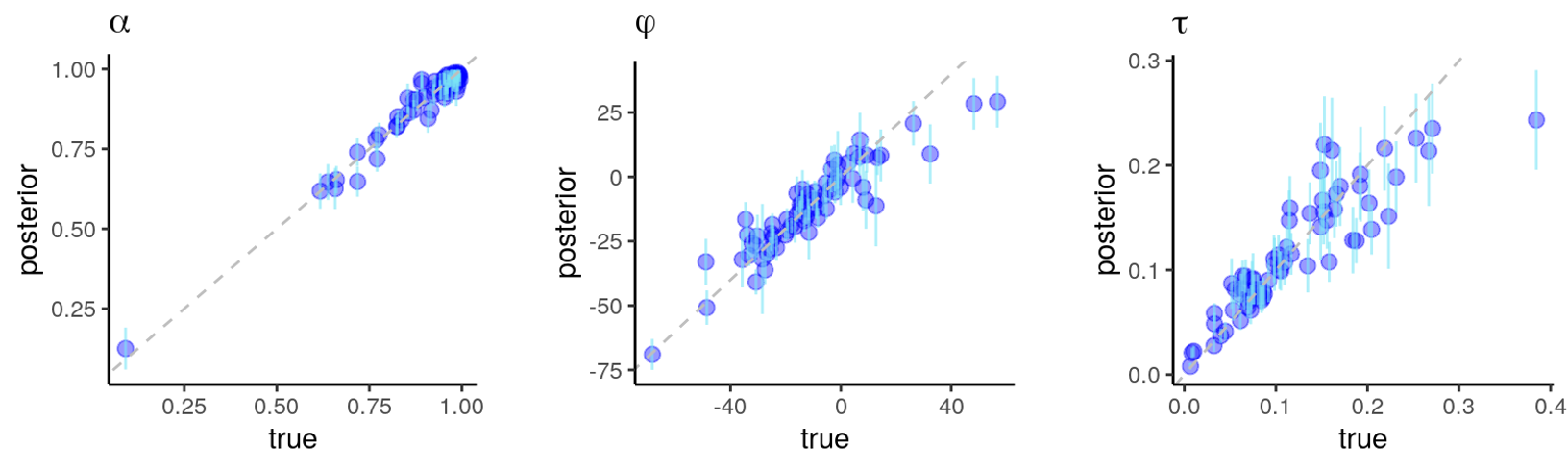
**

We ran a parameter recovery test to evaluate whether the parameters of our best-fitting model can be reliably identified from task behavior. Using the parameter estimates obtained from the empirical data as “ground-truth” values, we generated simulated choices and then re-estimated the parameters from those simulated data. We found a strong correspondence between the ground-truth values and the recovered estimates, indicating that the model parameters are reliably identifiable from choice behavior. All parameters demonstrated good recoverability, with recovered estimates closely tracking the ground-truth values (α: *r* = 0.98, *p* < .001; φ: *r* = 0.92, *p* < .001; τ: *r* = 0.90, *p* < .001). These results suggest that each parameter can be estimated robustly from observed choice data and captures a distinct computational contribution to behavior in the task. Each dot represents an individual participant’s ground-truth and recovered posterior parameter estimates, and the line indicates the corresponding credible interval.

**Supplementary Figure 5.** The relationship between model parameters and choice in the task using simulation



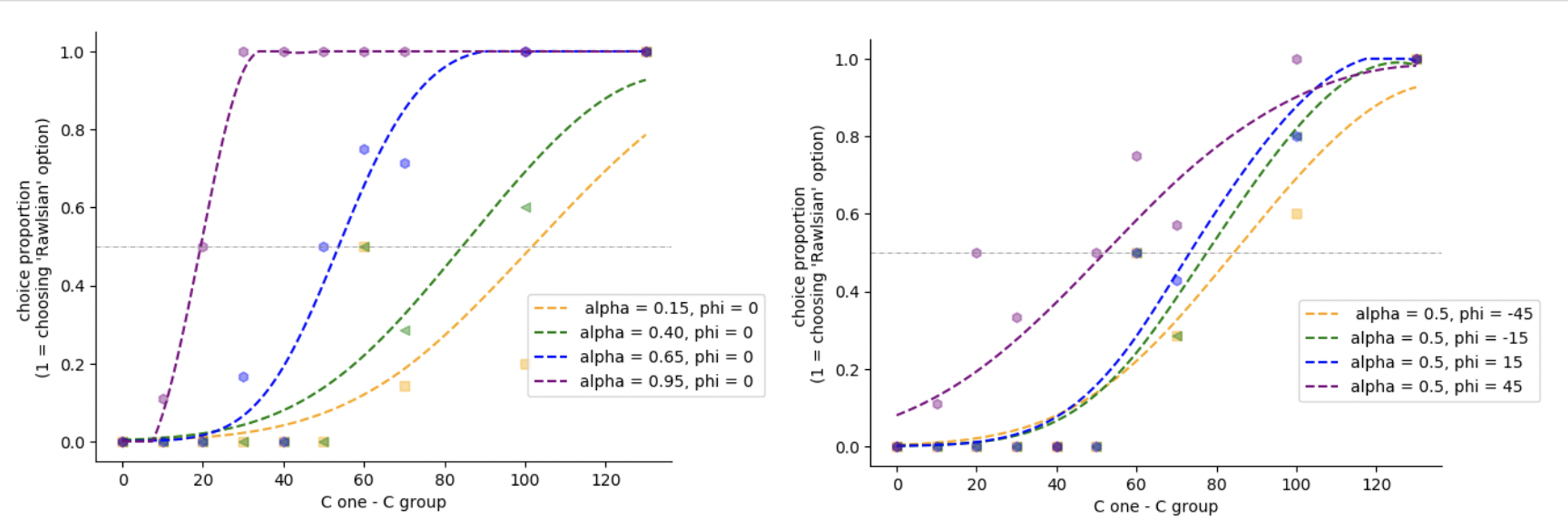


We generated the simulated choice data with the winning model by changing parameter values one at a time while fixing another to examine the respective roles of α and φ parameters on choices across our task parameter space. We fitted the simulated data to the cumulative normal distribution as a function of relative difference in the amount of harm for an individual when choosing the one compared to for choosing the group as in maximin computation ($C_{one}$ > $C_{group}$). This revealed that varying alpha in our model changed the slope of the cumulative normal distribution, modifying the sensitivity to the relative difference in harm given to the individual. On the other hand, Phi had an effect on adjusting the point of subjective equivalence, changing the point in which people regard fair to assign the harm more or less to a single individual.

**Supplementary Figure 6.** The relationship between phi parameters and choice in the task space using simulation


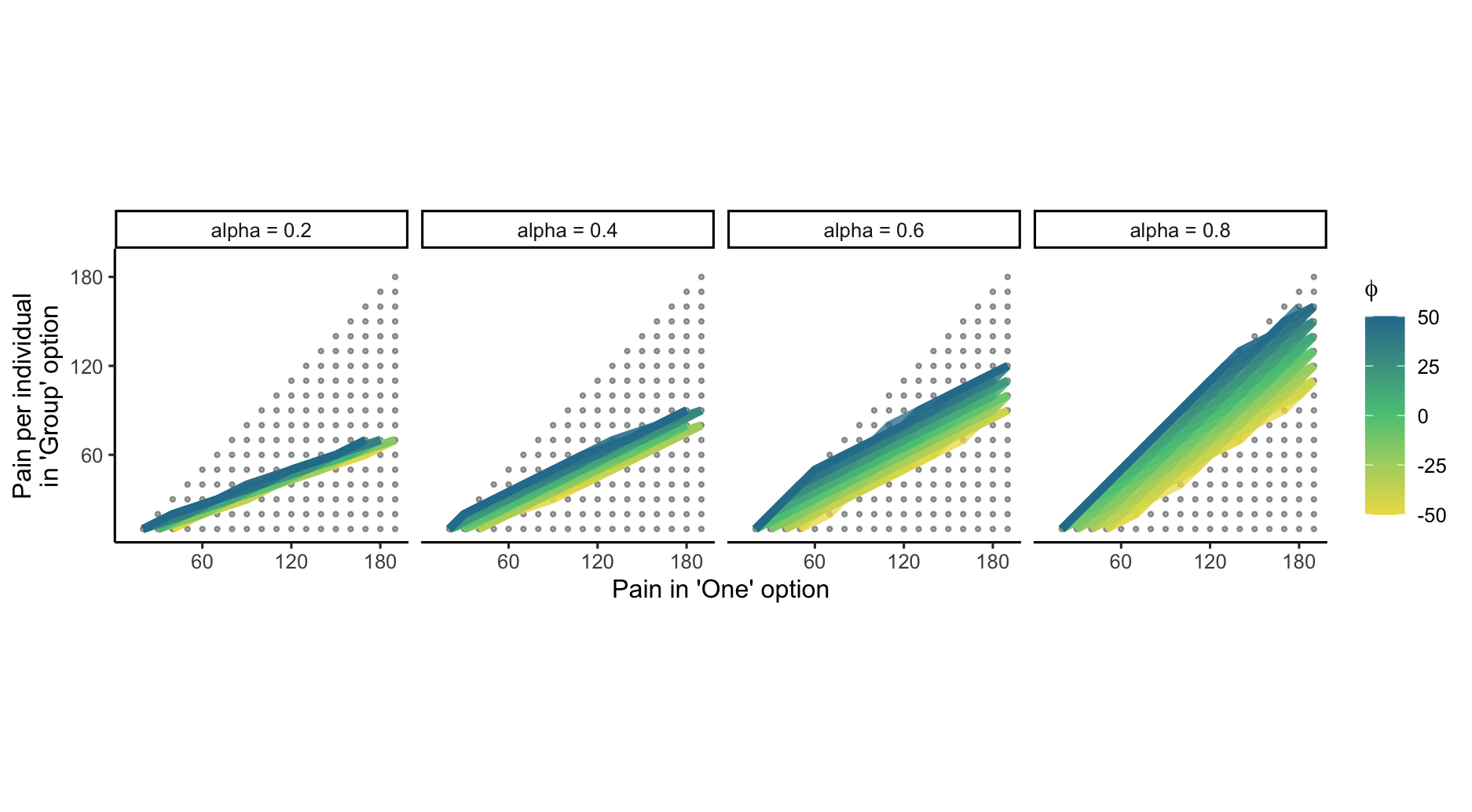


Simulations across the task parameter space were run over a range of $\varphi$ values while holding α fixed at a certain value. These simulations confirmed that increasing $\varphi$ systematically shifts the choice boundary in the direction that increases selection of the Rawlsian option across our parameter space of pain given to the “one” option vs per individual in the “group”. Note that we used an extended range of $\varphi$ values to make the effect visually more apparent.

**Supplementary Figure 7. Posterior density plots for parameter estimates**


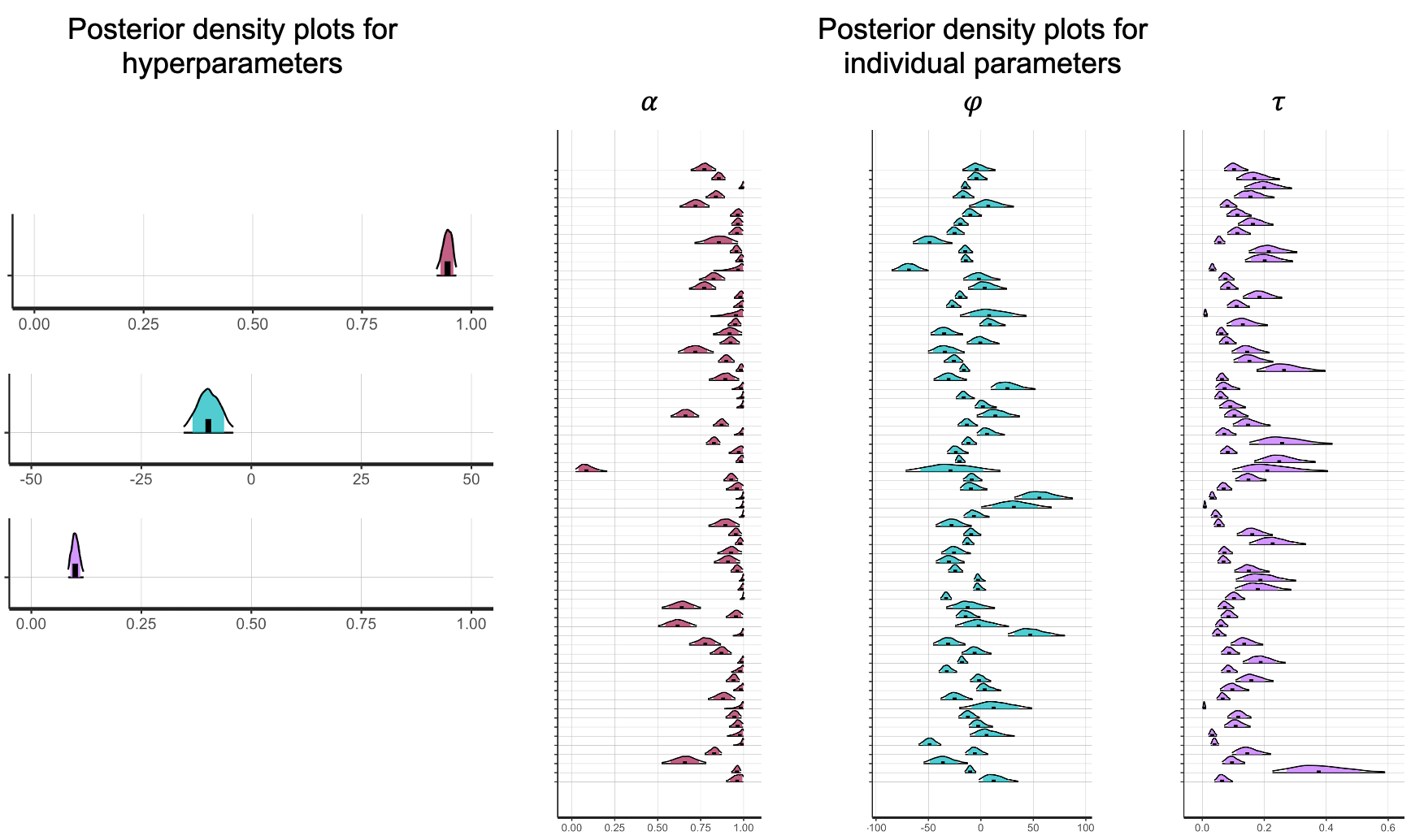


We report density plots of the credible intervals for parameter estimates at both the group level and the individual participant level. We also estimated the parameter values separately for each session and found high cross-session reliability (α: Cronbach’s α = 0.95, φ: Cronbach’s α = 0.85, τ: Cronbach’s α = 0.69).

**Supplementary Figure 8. Task parameter and Rawlsian choice ratio by group size**


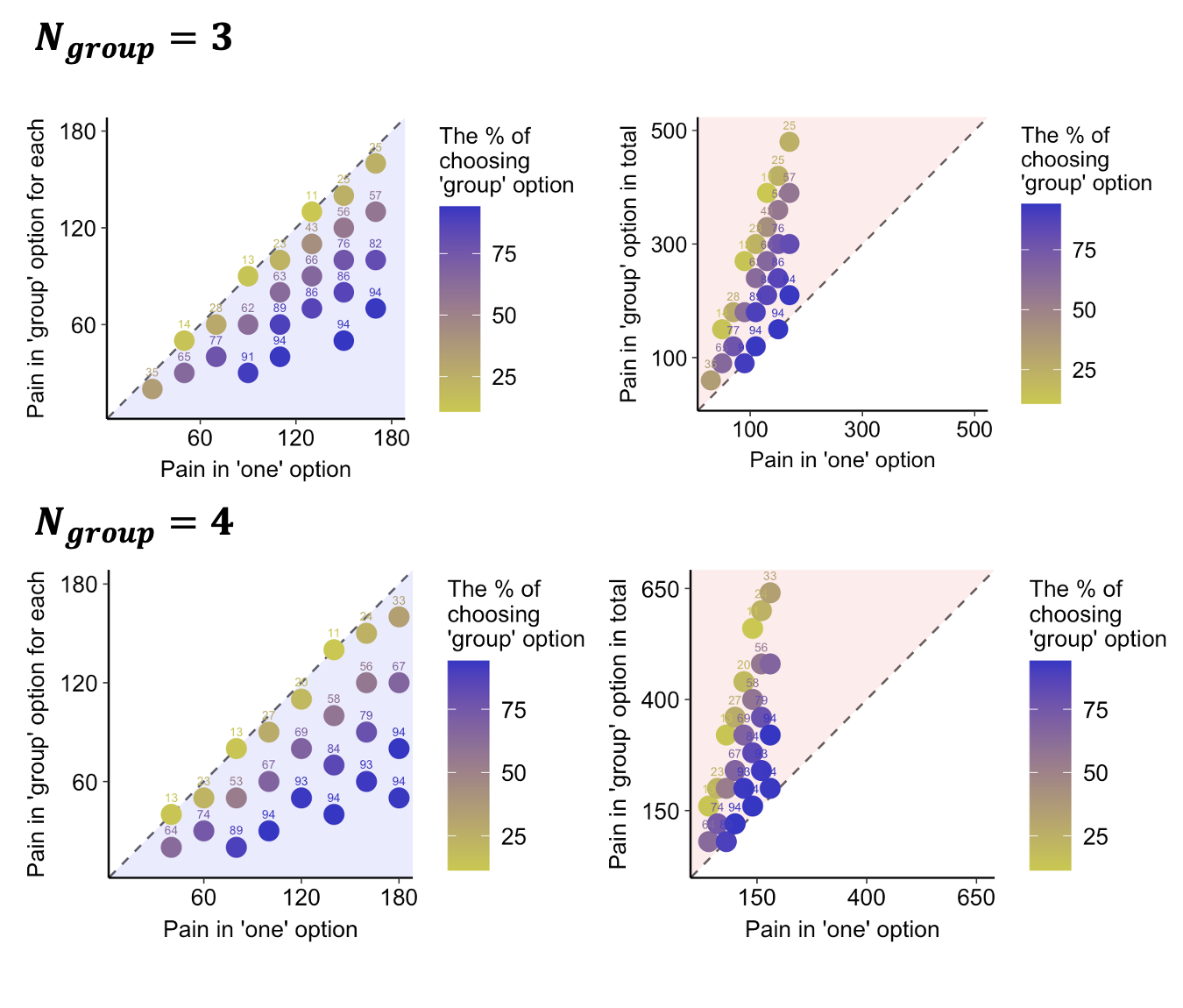

Choice of the Rawlsian (group) option across combinations of harm to the individual and harm to the group. Results are displayed separately by group size.

**Supplementary analysis**

**1.Participants’ choice preferences analyzed with mixed effects logistic regression**

To examine participants’ choice preferences, we also ran mixed-effects logistic regressions to account for the repeated-trials structure of the data and to assess whether the results support the same conclusions as the paired t-tests on choice proportions. As reported below, the mixed-effects analyses converge on the same conclusions: participants showed an overall preference for the Rawlsian option, and we found no evidence for an overall Kantian preference. Instead, participants tended to choose the Rawlsian option regardless of whether it was presented as the default.

1) Participants’ overall tendency to choose the Rawlsian option was analyzed using a mixed-effects logistic regression with a fixed intercept and a random intercept for participant (68 participants). The intercept was significantly greater than zero, indicating that participants chose the Rawlsian option more often than chance (β = 0.43, SE = 0.13, z = 3.46, p < .001), corresponding to an estimated choice probability of 0.61.

2) We tested whether switching away from the default option differed between the one-default and group-default conditions (excluding no-default trials) using a mixed-effects logistic regression with default condition (one-default vs. group-default) as a fixed effect and participant as a random intercept. The effect of the default condition was significant, indicating a higher probability of switching in the one-default condition (β = 0.76, SE = 0.05, z = 15.40, p < .001).

3) We tested whether the Rawlsian option was more likely to be chosen when it was presented as the default. Choices were analyzed with a mixed-effects logistic regression predicting Rawlsian choice from condition (group-default, one-default, no-default), with participant as a random intercept. Relative to the group-default condition (reference level), Rawlsian choice was less likely in the no-default condition (β = −0.21, SE = 0.05, z = −3.88, p < .001) and in the one-default condition (β = −0.24, SE = 0.05, z = −4.40, p < .001), indicating a higher probability of choosing the Rawlsian option when it was presented as the default.

**2. The relationship between participants’ beliefs about the procedure and their choices.**

We tested whether participants’ beliefs about the procedure influenced their decisions. We found no evidence of such an effect: participants’ belief measures were not correlated with choice ratio (*r* = 0.05, *p* = 0.687), α (*r* = 0.10, *p* = 0.438), or φ (*r* = -0.01, *p* = 0.971). We also compared the estimated α and φ parameters between participants with low versus high belief and observed no statistically significant differences between the groups (choice ratio: *t* = -0.09, *p* = 0.926; α: *t* = 0.33, *p* = 0.742 ; φ: *t* = -0.09, *p* = 0.928).

**3. PPI analysis**

1. **Functional connectivity responsive to moral decision**

Our results from parametric brain responses to maximin computation provided evidence that concern for the worst-off individual in harm allocation decision engages brain regions implicated in mentalizing. Moreover, individual differences in Rawlsian preference for prioritizing the reduction of the worst off individual’s suffering over aggregate utility was associated with differential neural sensitivity to the maximin computation, with neural activity in mentalizing and valuation network scaling with such preferences respectively in positive and negative directions.
 These findings motivated us to hypothesize that, given the role of vmPFC in encoding all-things-considered decision value (Shenhav & Greene, 2014; Hutcherson et al., 2015), individual differences in Rawlsian preference may be explained by the degree to which one incorporates concerns for the worst-off individual into integrative values of moral decision through mentalizing. This possibility is also consistent with previous works showing that social information represented in brain areas specialized for encoding social functions modulates computation of decision value in domain-general valuation regions (Izuma, Saito & Sadato, 2008; Lin, Adolphs & Rangel, 2011; Ruff & Fehr, 2014; Zoh, Chang, & Crockett, 2021). Therefore, we predicted that mentalizing region will express differential functional connectivity with vmPFC, during Rawlsian choice relative to uUtilitarian choice as a function of Rawlsian preference. To test this, we implemented psychophysiological interaction (PPI) analyses with vmPFC as a seed region and added individual differences in moral preferences as second (group) level regressor onto the contrast between the trials where Rawlsian option was chosen over Utilitarian one.
 When choosing Rawlsian option over Utilitarian one, participants with stronger Rawlsian preferences showed greater increase in connectivity between vmPFC and dmPFC that has been implicated in mentalizing. This supported our prediction that Rawlsian preference is explained by the degree to which one engages in mentalizing during moral decision-making, through which they may incorporate concerns for the worst-off individual in their decisions. Interestingly, we also found that, even in the case of making Utilitarian option over Rawlisan option, Rawlsian preference was associated with greater increase in connectivity between vmPFC and TPJ and IPL, areas of which also encompass mentalizing network (Saxe, 2006; Koster-Hale & Saxe, 2013; Jamali et al., 2021; Schurz et al., 2014; Schurz et al., 2021). Together, this suggests that Rawlsian preference is associated with greater engagement of mentalizing region in the brain, regardless of choices participants made.

**
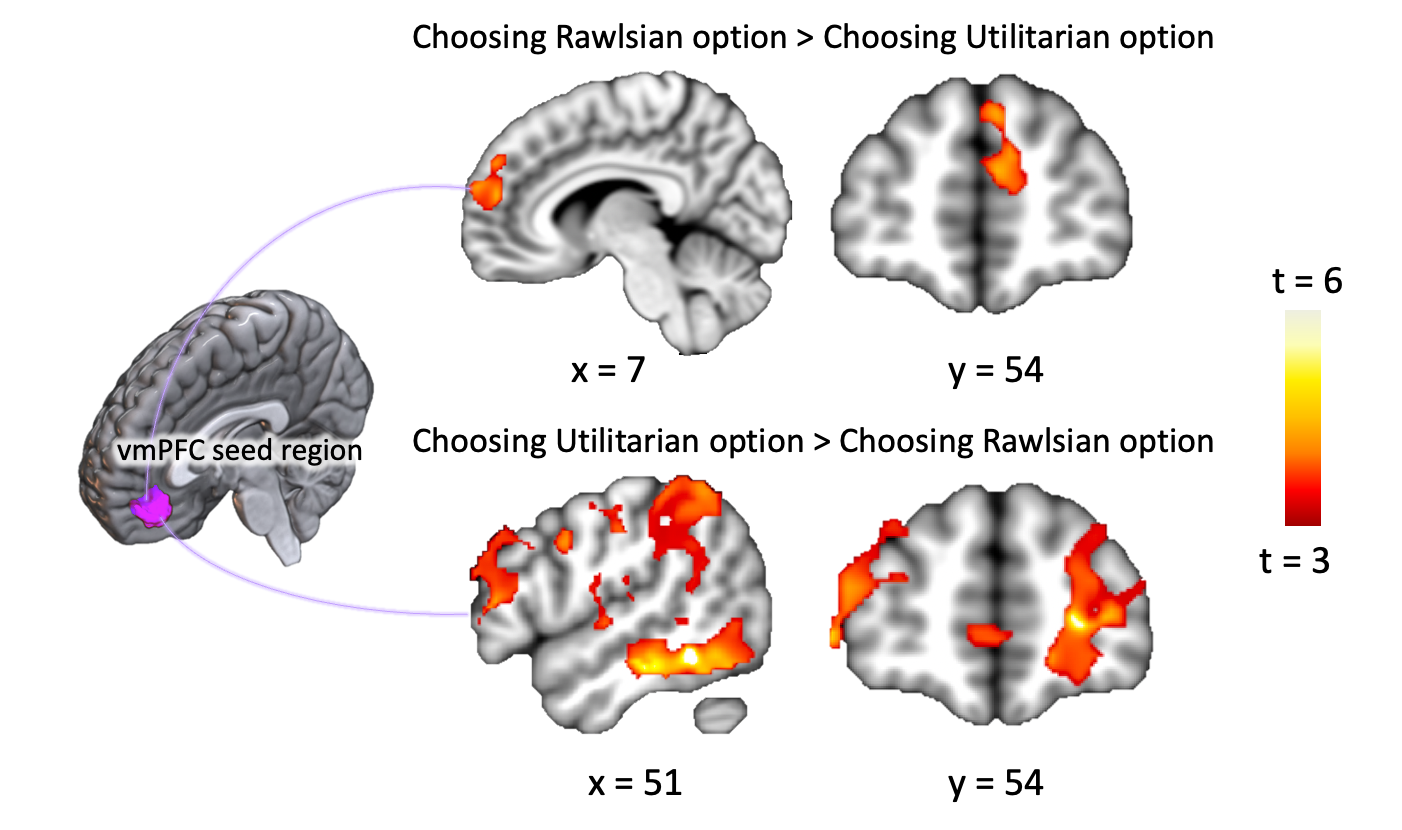
**

Heightened functional connectivity between vMPFC and a set of brain regions in mentalizing regions as a function of choice type was modulated by individual’s Rawlsian moral preference. People with higher Rawlsian preference showed the greater increase in connectivity between dmPFC and vmPFC when making Rawlsian choice compared to Utilitarian choice. Rawlsian preference was also associated with heightened connectivity between vmPFC and brain regions in mentalizing regions when making utilitarian choice compared to Rawlsian choice.

1. **PPI model: functional connectivity with vmPFC**

We applied the method of generalized psychophysiological interactions (gPPI, McLaren et al., 2012) to determine brain regions with which differential functional connectivity with vmPFC as a function of choice type (choosing Rawlsian option > Utilitarian option) is modulated by individual differences in Rawlsian preference. Seven participants were excluded from PPI analysis, since they did not provide sufficient variation in their choices to allow for contrast estimation. For the gPPI analysis, we first constructed vmPFC region-of-interest (ROI) by masking a contrast from GLM2, which showed parametric effects of relative chosen value, with a *priori* meta-analysis map of vmPFC carrying a SV signal (5-way conjunction analysis map) in Bartra et al., (2013). Next, we built a gPPI model which contained PPI regressors for the event of choosing Rawlsian option and choosing Utilitarian option. The model also contained five additional event regressors of no interest for each phase of the task. The contrast of choosing Rawlsian option compared to Utilitarian option was calculated using the model. We regressed parameter alpha onto the contrast to look for brain regions with which they showed increased functional coupling during the decision onset of the trial where Rawlsian option was chosen relative to the Utilitarian option. All the results with univariate fmri analysis reported in the text survived whole-brain correction for multiple comparisons (P < 0.05, FWE-corrected at the cluster level after voxel-wise thresholding at P < 0.001)
